# Human cardiac measurements with diamond magnetometers

**DOI:** 10.1126/sciadv.aeg5281

**Published:** 2026-09-16

**Authors:** Muhib Omar, Magnus Benke, Shaowen Zhang, Jixing Zhang, Yihua Wang, Michael Kuebler, Pouya Sharbati, Ara Rahimpour, Arno Gück, Maryna Kapitonova, Devyani Kadam, Carlos René Izquierdo Geiser, Jens Haller, Arno Trautmann, Katharina Jag-Lauber, Robert Rölver, Thanh-Duc Nguyen, Leonardo Gizzi, Michelle Schweizer, Mena Abdelsayed, Ingo Wickenbrock, Andrew M. Edmonds, Matthew Markham, Peter A. Koss, Oliver Schnell, Ulrich G. Hofmann, Tonio Ball, Jürgen Beck, Dmitry Budker, Joerg Wrachtrup, Arne Wickenbrock

**Affiliations:** ^1^ Helmholtz-Institut Mainz, GSI Helmholtzzentrum für Schwerionenforschung, Staudingerweg 18, Mainz 55128, Germany.; ^2^ Johannes Gutenberg-Universität Mainz, Saarstr. 21, Mainz 55122, Germany.; ^3^ 3rd Institute of Physics, Zentrum für Angewandte Quantuntechnologie (ZAQuant), University of Stuttgart, Allmandring 13, Stuttgart 70569, Germany.; ^4^ Neuromedical AI Lab, Department of Neurosurgery, Medical Center–University of Freiburg, Faculty of Medicine, University of Freiburg, Freiburg, Germany.; ^5^ Department of Physics, University of California, 366 Physics North, MC 7300, Berkeley 94720, CA, USA.; ^6^ Fraunhofer Institute for Physical Measurement Techniques (IPM), Georges-Köhler-Allee 301, Freiburg 79110, Germany.; ^7^ Medical Faculty, University of Freiburg, Hugstetter Str. 55, Freiburg im Breisgau 79106, Germany.; ^8^ Department of Neurosurgery, Medical Center, University of Freiburg, Breisacher Str. 64, Freiburg im Breisgau 79106, Germany.; ^9^ Q.ANT GmbH, Handwerkstraße 29, Stuttgart 70565, Germany.; ^10^ Fraunhofer Institute for Production Engineering and Automation, Nobelstraße 12, Stuttgart 70569, Germany.; ^11^ Lankenau Institute for Medical Research, Penn Wynne, PA, USA.; ^12^ Medizinische Klinik I, Abteilung für Kardiologie, Elektrophysiologie, Pneumologie und konservative Intensivmedizin, Klinikum Lünen, Altstadtstraße 23, Lünen 44534, Germany.; ^13^ Element Six Global Innovation Centre, Fermi Avenue, Harwell Oxford, Didcot, Oxfordshire OX11 0QR, UK.; ^14^ Department of Neurosurgery, Uniklinikum Erlangen, Friedrich-Alexander-Universität Erlangen-Nürnberg, Hugenottenpl. 6, Erlangen 91054, Germany.; ^15^ Center for Advanced Surgical Tissue Analysis (CAST), Faculty of Medicine, University of Freiburg, Freiburg, Germany.; ^16^ Institute for Machine-Brain Interfacing Technology, University of Freiburg, Freiburg, Germany.

## Abstract

The heart produces small magnetic signals, but detecting them without touching the body and without heavy shielding equipment remains a major challenge. In this study, we demonstrate a noninvasive method to measure these signals using compact quantum sensors made from diamond. These sensors operate at room temperature and can detect extremely weak magnetic fields produced by the heart, even outside highly shielded environments. By averaging signals over many heartbeats and using noise suppression techniques, we successfully recorded clear magnetocardiography traces. Although current measurements require averaging, our results show a clear path toward real-time detection. We also explored a gradiometry protocol for unshielded noisy environments, paving the way for contactless cardiac detection in challenging clinical conditions. This work points toward a path for using quantum sensors in practical medical diagnostics of the heart and brain.

## INTRODUCTION

Weak magnetic fields generated by neuronal and muscular activity, especially cardiac magnetic fields, carry rich physiological information. The quantum mechanical properties of electron spin can facilitate the detection of such signals. From nuclear magnetic resonance to atomic sensors to superconducting quantum interference devices (SQUIDs), nuclear and electron spins have enabled some of the most sensitive probes of nature. Today, advances in spin-based quantum sensors are opening new pathways for noninvasive biomedical diagnostics. Here, we report magnetocardiography (MCG), i.e., measurement of magnetic fields produced by cardiac electrical activity using diamond electron spin–based devices.

Electrocardiography (ECG) and MCG both rely on cardiac electrical activity, with the former recording electric potential differences and the latter recording magnetic fields, reflecting the same physiological processes of depolarization and repolarization. ECG signals originate from action potentials in the heart, and clinicians can record them on the body surface through electrodes. This technique is mature, low cost, and widely available, making it the clinical gold standard for routine diagnosis. However, the conductivity of body tissues strongly influences ECG signals, resulting in limited spatial resolution, while the necessity of skin contact precludes application in situations where contact is not possible (e.g., burn victims).

In contrast, MCG signals arise from magnetic fields generated by cardiac currents, and highly sensitive magnetometers can detect them. Clinicians can use MCG signals to diagnose conditions such as arrhythmia and coronary artery disease (*1*, *2*). As a noncontact technique, tissue conductivity minimally affects MCG, enabling the detection of subtle abnormalities that ECG may miss and support precise localization of pathological sources (*1*). Historically, MCG relied on SQUIDs, whose operation exploits macroscopic quantum coherence in superconductors. More recently, optically pumped magnetometers (OPMs), based on atomic spin polarization in vapor cells, have emerged as an alternative. Other sensor technologies, such as fluxgate and magnetoresistive sensors, have also demonstrated MCG detection (*3*, *4*). Despite its clinical promise, MCG faces major challenges: Cardiac magnetic signals are inherently weak, environmental magnetic noise highly affects measurements, and current systems typically require stringent magnetic shielding. High system complexity and cost have further limited widespread clinical adoption. Researchers therefore best view ECG and MCG as complementary modalities (see Table 1), whose integration may substantially improve early diagnosis and spatial localization of cardiac pathologies.

**Table 1.** Comparison of several electric and magnetic heart signal detection technologies. Comparison of ECG and MCG technologies with different sensing modalities. This overview contextualizes the clinical application of MCG relative to ECG and positions diamond-based sensors within the broader landscape of established MCG methodologies.

	ECG	MCG		
Quantum sensor		SQUID	OPM	Diamond
Principle	Measures electric potentials	Measure magnetic fields		
Signal	Electric field, strongly affected by tissue conductivity (lungs, fat, fluid distort signals)	Magnetic field, hardly attenuated by tissues, minimally affected by conductivity distribution		
Contact method	Attached to the body surface	Noncontact		
		Safety distance due to cryostat	Safety distance due to cell temperature	Skin contact
Spatial resolution	Centimeter level	High, enabling precise localization and imaging		
		Millimeter level	Millimeter level	Micrometer level
Advantages	Mature, inexpensive	Noncontact, high patient comfort		
	Easy to operate, widely available	Unaffected by tissue conductivity		
	Suitable for dynamic monitoring (long-term, exercise ECG)	Detects subtle or deep abnormalities, useful for arrhythmia source localization and early ischemia detection, access to fetal MCG		
Disadvantages	Strongly influenced by tissue properties	Expensive		
	Limited localization ability	Substantial environmental constraints requiring a shielded room or gradiometric schemes		
	Low sensitivity to early or subtle abnormalities	Not yet widely adopted in clinical practice		
Application scenarios	Arrhythmia	Arrhythmia origin localization		
	Myocardial infarction	Detection of microischemia		
	Routine clinical examinations	Electronic pathway reconstruction		
		Fetal MCG		
Popularity	Established as the clinical “gold standard”	Mainly used in research and specialized clinical centers		

Current state-of-the-art MCG systems rely predominantly on SQUIDs (*5*), which require cryogenic cooling and thus hinder portability and broad clinical deployment. OPM-based systems have demonstrated operation in unshielded environments (*6*), but they face challenges related to heading errors, dead zones, and elevated operating temperatures that complicate contact-based measurements (*7*–*9*). Consequently, researchers still regard the development of miniaturized, room-temperature MCG technologies capable of providing full vector information in unshielded environments as a key goal in biomagnetic sensing.

One promising pathway toward this goal relies on nitrogen-vacancy (NV) centers in diamond, a solid-state spin system that has emerged as a leading platform in quantum sensing. Over the past two decades, researchers have widely used NV centers to measure temperature (*10*), strain (*11*), electric fields (*12*), magnetic fields (*13*), and rotation (*14*). NV magnetometry directly exploits the coherent manipulation and optical readout of individual and ensemble electron spins at room temperature, exemplifying how fundamental spin physics has transitioned into practical sensing technologies. Compared with OPMs, NV magnetometers feature a much smaller sensing volume, facilitating gradiometer-based and vector-resolved MCG measurements under unshielded conditions. Their comparatively high volume sensitivity (*15*) makes them competitive with OPMs when combined with flux concentrators (*16*). The lower intrinsic sensitivity of NV sensors can even be advantageous, as it prevents flux concentrator noise from becoming a dominant limitation (*17*). NV magnetometers further offer fast initialization, excellent biocompatibility, and stable operation over a broad temperature range, making them particularly attractive for biomedical applications. Researchers have already demonstrated proof-of-principle NV-based MCG detection in animal studies, both invasively (*18*) and noninvasively using flux concentrators (*19*).

In this study, we report the detection of human cardiac magnetic signals using NV magnetometers independently developed by three groups: Johannes Gutenberg University Mainz (JGU), the University of Stuttgart (ZAQuant), and the quantum technology start-up Q.ANT GmbH. These results mark a important milestone in the translation of spin-based quantum sensors from laboratory demonstrations to real-world biomedical applications.

They demonstrate that, a century after the discovery of spin, NV diamond magnetometry has matured to the point where researchers can measure meaningful biomagnetic signals under diverse experimental conditions. This progress enables a quantitative assessment of the remaining signal-to-noise ratio (SNR) requirements and positions NV-based MCG as a competitive and potentially more accessible alternative to existing technologies, with long-term prospects for both clinical and home-based cardiac monitoring.

## RESULTS

This section describes the three integrated NV magnetometers and the corresponding MCG measurement procedures, covering both zero– and finite–bias field configurations operated in shielded, partially shielded, and unshielded environments. It further reports a cross-validation of the NV-based MCG signals using a commercial OPM sensor array and concludes with gradiometric noise suppression results that demonstrate the feasibility of NV magnetometry in realistic, noisy, and unshielded settings.

### MCG with NV-based magnetometers

**Fig. 1.**
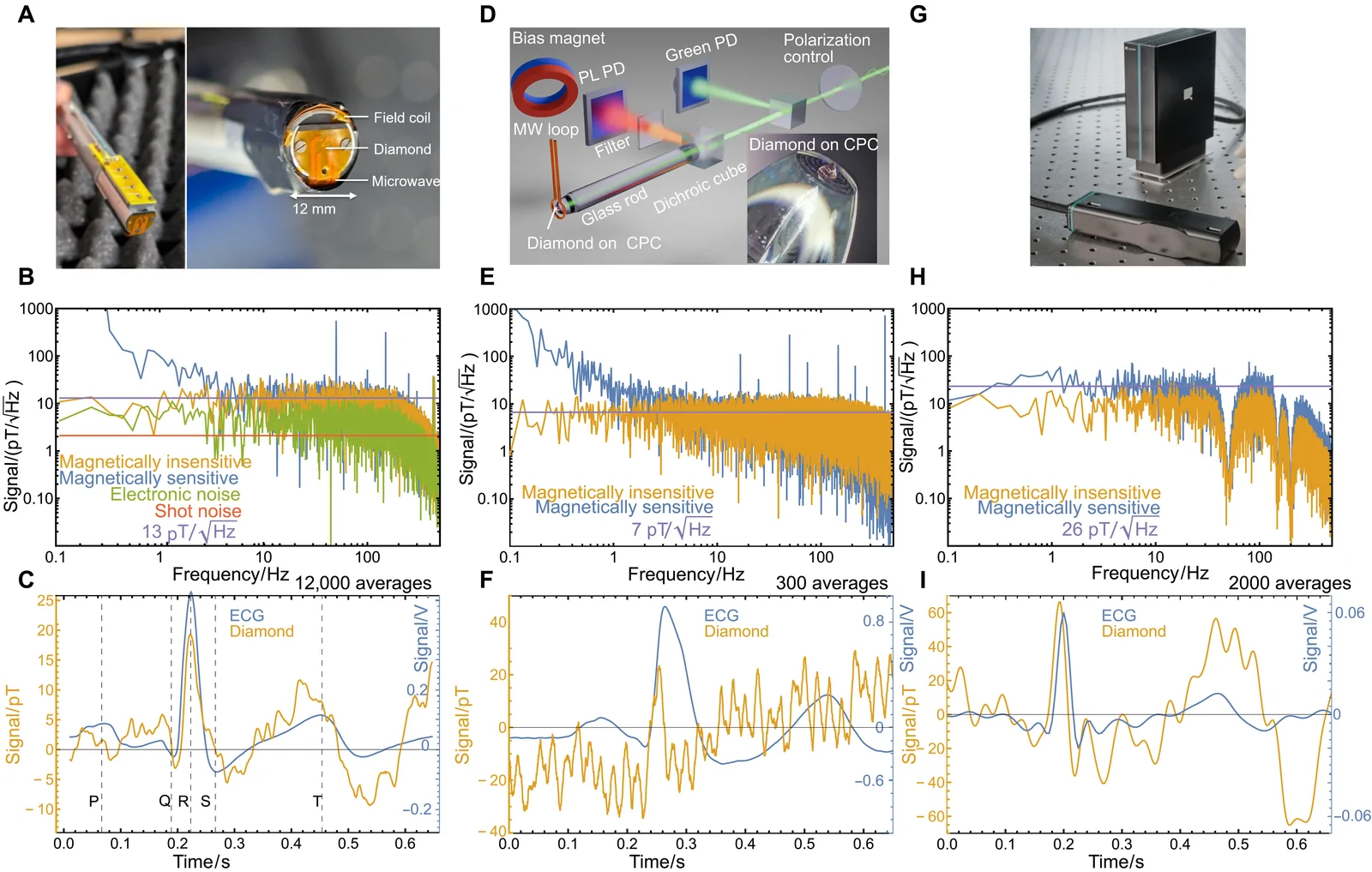
NV-based magnetic sensors, sensor characterization, and detected MCG signals. First column: JGU. (**A**) Diamond sensor head dimensions. (**B**) Amplitude spectral density with a baseline of 13 pT/$\sqrt{\text{Hz}}$, measured inside the shielded room of the Helmholtz Institute Mainz (JGU). (**C**) ECG trace and diamond MCG signal (12,000 averages) with indications of the main components of the ECG, P wave, QRS complex, and T wave. Second column: ZAQuant. (**D**) Rendering of the sensor, created in Blender (*45*). PD, photodiode; PL, photoluminescence. For a description of the parts refer to the main text. (**E**) Measured amplitude spectral densities inside the partially shielded environment, showing a noise baseline of 7 pT/$\sqrt{\text{Hz}}$. (**F**) ECG trace and averaged diamond MCG signal (300 averages). Third column: Q.ANT. (**G**) Sensor design. (**H**) Amplitude spectral density with a baseline of 26 pT/$\sqrt{\text{Hz}}$; filters were applied during acquisition. (**I**) ECG trace and diamond MCG signal (2000 averages).

We first describe the JGU MCG measurements conducted at the Helmholtz Institute Mainz within its multilayer magnetically shielded room using the zero bias–field NV magnetometer. Afterward, the MCG measurements realized with bias field NV magnetometers at the University of Stuttgart and at Q.ANT GmbH are introduced. Schematics of the sensors, their noise characteristics, and the MCG measurements for all sensors can be seen in Fig. 1.

**Fig. 2.**
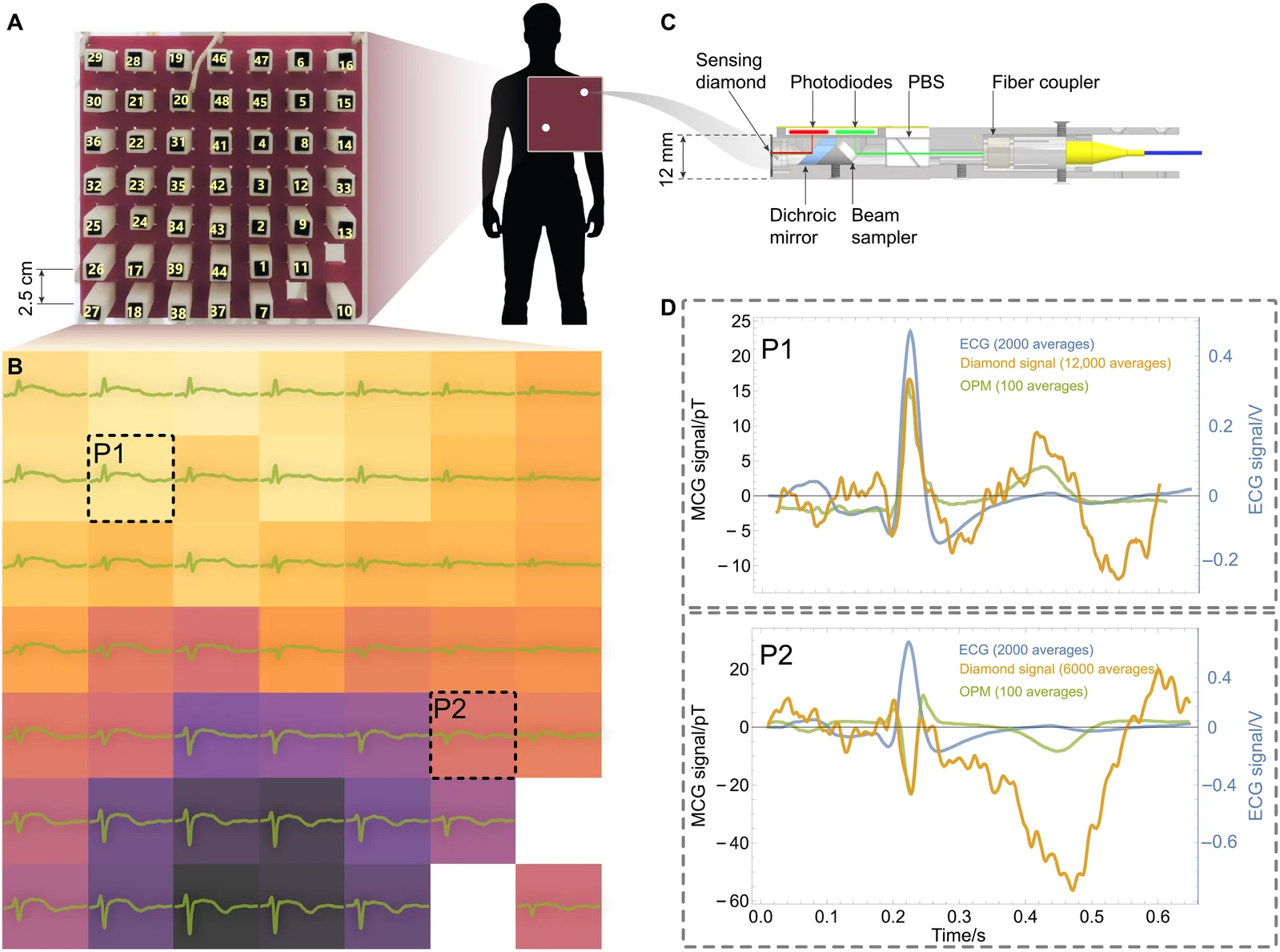
Validation measurements of the MCG signal detected by a diamond sensor using an OPM array. (**A**) OPM array with sensor labeling and its position on the individual’s chest. The position of the diamond sensor measurements are indicated as the white dots on the torso. Figure prepared using Inkscape (*46*). (**B**) The 47 different OPM MCG signals after band-pass filtering and averaging 100 times. The color overlay indicates the amplitude of the QRS complex. P1 and P2 mark the positions of the diamond sensor measurements. (**C**) Schematic of the JGU sensor (photographs shown in Fig. 1A) with indication of the main components. Working principle and more details can be found in (*21*). PBS, polarizing beam splitter. (**D**) The resulting OPM, ECG, and diamond signal traces for MCG signal detection at the positions of P1 and P2 with respect to the OPM array.

To record human cardiac magnetic signals in Mainz, we used a fiberized NV diamond magnetometer designed for the use as a transportable endoscope. The sensor is operated without a magnetic bias field. The sensing protocol using an oscillating magnetic field to recover magnetic sensitivity without a dc bias magnet is described in (*20*, *21*). Zero-field shielded environments are straightforward to create and minimize bias field fluctuations and magnetic field gradients (*22*). This makes them compatible with existing setups for OPMs used to validate the MCG detection (see the “Validating the MCG signature” section). The sensing diamond (*23*) was a truncated pyramid with 0.18 mm in height and a 0.5 mm–by–0.5 mm base with a (100)-oriented top surface. A magnetic sensitive noise level of 13 $\text{pT}/\sqrt{\text{Hz}}$ (averaged between 20 and 30 Hz) was achieved in the magnetically shielded room (compare Fig. 1B). This value was based on individual time-series measurements of 9-s duration, processed using a Hann window before Fourier transformation. The amplitude spectrum was then divided by the square root of the effective noise bandwidth (*24*) to obtain the amplitude spectral density. Magnetically insensitive traces were acquired under sensor operating conditions that closely matched those used for magnetically sensitive measurements but with the sensor-specific magnetic readout mechanism disabled. For the Mainz sensor, the magnetic field modulation was turned off. For the Q.ANT and ZAQuant sensors, the microwave (MW) power was turned off. Electronic noise traces were also recorded with the laser light turned off. The magnetically insensitive noise floor indicated residual laser noise as the limiting noise source. A detailed discussion of the noise floor characteristics and sources can be found in (*21*). The endoscopic sensor design is described in detail in (*21*, *25*) and shown in Figs. 1 (A to C) and 2C with the sensor head components. During data collection, the sensor was positioned ∼1 cm away from a seated individual’s chest, leaving sufficient space for breathing. Data were recorded in 22-min intervals. Electrodes were placed on the individual’s back to record reference ECGs used as a trigger to average the data across multiple cardiac cycles to account for heart rate variability (*26*). Averaging was necessary since magnetic field recordings did not reveal the MCG signal in the NV trace in real time.

For scalable bedside MCG applications in unshielded settings, bias field NV magnetometers could be advantageous. In addition to requiring a stable bias magnetic field, they must also suppress ambient magnetic noise, which poses a major challenge in real-world environments. The sensors from ZAQuant and Q.ANT followed this route. These sensors are presented in Fig. 1 (D and G). As depicted for the ZAQuant sensor, the laser light enters the image from the right side. The power onto the diamond is adjusted by a polarizer and a polarizing beam splitter (not shown in the image for visual clarity). To control the polarization afterward a λ/2 wave plate is used. The diverging beam is collimated by lenses (again omitted in the image for visual clarity) and brought to the light guidance optics. To account for laser power noise, we split part of the laser beam by a 90/10 beam splitter and measured it by a reference photodiode. The endoscope-like light guidance optics consist of a cubic beam splitter with a dichroic coating, a cylindrical light pipe, and a compound parabolic concentrator (CPC) lens. The pyramid-shaped diamond sits on top of the CPC lens, as depicted in the inset of Fig. 1D. All parts are glued together with an adhesive photoresist. The generated fluorescence is guided through the same optical system and a 695-nm long-pass filter onto the measurement photodiode. The MW is delivered through a single loop antenna, and a ring permanent magnet produces the magnetic bias field, which is mounted in a custom three-dimensional (3D) printed holder and aligned to the (100) axis of the diamond. For a more detailed explanation of the chosen parts and their optimization, see (*25*). The sensitivity analysis was carried out analogously to the zero-field NV sensor, the only difference being that the time trace durations were 60 s (ZAQuant) and 10 s (Q.ANT). Sensitivities of 7 $\text{pT}/\sqrt{\text{Hz}}$ (ZAQuant) and 26 $\text{pT}/\sqrt{\text{Hz}}$ (Q.ANT) were determined. For the MCG measurements at ZAQuant, the individual was sitting 1 cm away from the sensor head. The Q.ANT measurements were performed with an individual lying directly below the sensor. The magnetic signal was recorded simultaneously with ECG to enable averaging. The corresponding MCG data and noise spectral density were subjected to a band-stop filter of a quality factor of 30 at the frequencies of 50, 150, and 200 Hz. Those were found to be the dominant line frequencies picked up at the unshielded measurement location. In addition, the data were high pass filtered above 8 Hz to avoid the large 1/*f* noise contribution from the unshielded magnetic environment dominating the signal. The MCG data from the ZAQuant measurements were filtered with narrow band-stop filters around 50 Hz, 60 Hz, and their harmonics to reduce the noise of these common interferences.

Recordings showed MCG results for each of the sensors. The amount of averages for the displayed data was 12,000, 300, and 2000 for JGU, ZAQuant, and Q.ANT, respectively. The number of averaged cardiac cycles was adjusted for each dataset based on recording duration, ECG stability, sensor positioning, and site-specific magnetic noise levels. Consequently, the averaging count is not a direct metric of intrinsic sensor sensitivity. Because correlated low-frequency magnetic noise and baseline drifts are only weakly suppressed by temporal averaging, using an identical number of averages across different sites or measurement geometries would not result in comparable waveform quality. Instead, the averaged traces serve primarily to identify reproducible, ECG-locked MCG features, while sensor sensitivities are evaluated independently using measured noise spectral densities.

The variance in SNR observed across Fig. 1 (C, F, and I) is primarily attributed to differences in the ambient magnetic environments rather than inherent disparities in single-shot sensor sensitivity. While all three sensors exhibit comparable intrinsic noise floors, the effective SNR is constrained by the local magnetic noise spectral density. In environments dominated by low-frequency 1/f noise, the efficacy of signal averaging is fundamentally limited by the temporal correlation of the noise, in contrast to white-noise sources (e.g., shot noise), which are efficiently suppressed through integration. Consequently, the varying levels of ambient magnetic interference at each respective measurement site dictate the achievable trace clarity and the apparent differences in the averaged signal plots. The varying sensitivities of the three NV-diamond magnetometers arise from differences in diamond geometry, optical and MW implementation, readout protocols, sensor head integration, and operating environments. The JGU system uses a 0.5 mm–by–0.5 mm–by–0.18 mm diamond housed in a compact 12-mm-diameter sensor head; it operates at zero dc magnetic bias using magnetic field modulation. The ZAQuant system uses a 0.5 mm–by–0.5 mm–by–0.5 mm diamond within a platform-style optical implementation, operating at a bias of ∼1 mT via MW frequency modulation. The Q.ANT system uses a larger 1 mm–by–1 mm–by–0.5 mm diamond in a highly integrated, packaged sensor head and similarly operates at a ≈1-mT bias with MW amplitude modulation; during these measurements, it was operated with an optical pump power of ∼100 mW. These discrepancies in design and operation directly influence the detected fluorescence levels, spin resonance contrast and linewidths, MW field uniformity, technical noise, and thermal/mechanical stability, as well as the systems’ susceptibility to magnetic field, temperature, and alignment drifts. Consequently, a larger diamond or sensing volume does not, in isolation, guarantee a lower magnetic noise floor. The reported sensitivities reflect the aggregate response and noise performance of each complete magnetometer implementation rather than any single intrinsic parameter of the diamond material.

### Validating the MCG signature

The JGU MCG signal was visible with the diamond sensor after averaging, triggering with the ECG signal from an electrode on the individual’s back. To be certain that the electrical signal from the heart was not accidentally picked up by the measurement electronics of the diamond sensor, we recorded the diamond MCG at two positions with different MCG peak polarity with the same ECG setting. This ruled out electronic cross-talk. To inform the diamond sensor positions and estimate the expected MCG signal amplitudes, we mapped the MCG signal over the chest area of the individual using a 49-channel FieldLine OPM array in a magnetically shielded room (two-layer μ-metal shielding) at the Fraunhofer Institute for Physical Measurement Techniques in Freiburg. The OPM array consisted of compact spin-exchange relaxation-free OPMs based on heated ^87^Rb vapor cells. FieldLine OPMs have a nominal sensitivity of ∼15 $\mathrm{fT}/\sqrt{\mathrm{Hz}}$ and compact sensor heads of about 13 mm by 15 mm by 30 mm (*27*).

The array sensor positions (see Fig. 2A) were indicated on form-fitting shirts to identify the positions for the diamond sensor measurement conducted afterward. The sensor spacing in the array is 2.5 cm, and the stand-off distance to the chest was at least 5 cm for all sensors. The array’s MCG signals were visible in real time. The OPM array data were acquired during a ∼5-min period. The OPM array MCG traces in Fig. 2B show the data from all sensors averaged over 100 heartbeats. Before averaging the OPM signals, a digital band-pass filter (pass band between 3 and 30 Hz) was applied to the data. The MCG peak was detected and used to align individual time traces for averaging.

The experimental arrangement for the diamond sensor MCG measurements is illustrated in Fig. 2C. Data were acquired at positions P1 and P2, with averaging performed over 12,000 and 6000 cardiac cycles, respectively. This disparity in averaging counts reflects the differences in available recording durations and the specific objectives for each dataset: Measurements at position P1 served as the primary basis for the diamond-OPM comparison, whereas the 6000-heartbeat average at position P2 was sufficient to verify the expected position-dependent reversal in MCG polarity under identical ECG-triggering conditions. The resulting averaged traces are compared against the corresponding OPM data and the reference ECG signal in Fig. 2D. The measured MCG peak amplitudes between OPM and diamond sensor were comparable and consistent with values in the literature (*28*–*30*).

Despite the differences in offset distance, sensitive volume, and sensitivity axis orientation of the respective sensors to the chest, the signals of both sensors coincidentally had matching *R* peak amplitudes but differed notably in the T wave shape. The time durations that are conventionally known as belonging to the P wave, QRS complex, and the T wave are indicated in Fig. 1C. Each duration, size, shape, and variation of P wave, QRS complex, and T wave over time reflect a specific phase in the cardiac dynamic cycle (*31*). This can be caused by positioning and sensing volume differences. The OPM arrays’ MCG signatures for each position show a large variability in the T wave signal around the measured position consistent with literature [e.g. (*5*)]. Position variation and variation in the orientation of the sensitive axis between OPM and diamond sensor can lead to substantial variation in T wave size and shape. Furthermore, the difference in sensitive volumes of the diamond and OPM sensors also affects the recorded T wave morphology due to signal differences and directional variation over the sensing volume and the distance to the source.

### Gradiometry performance

Gradiometric configurations mitigate a key challenge in biomagnetic sensing: strong and spatially uniform environmental noise, which, in clinical or laboratory environments, can exceed the amplitude of cardiac signals by several orders of magnitude. By measuring the field difference between two spatially separated sensors, a gradiometer extracts the local field gradient. This differential measurement suppresses common-mode magnetic noise while preserving spatially localized biomagnetic signals. In practice, a well-matched gradiometer can achieve common-mode rejection ratios exceeding 60 dB, equivalent to a suppression factor of 1000 for homogeneous noise fields (*32*).

**Fig. 3.**
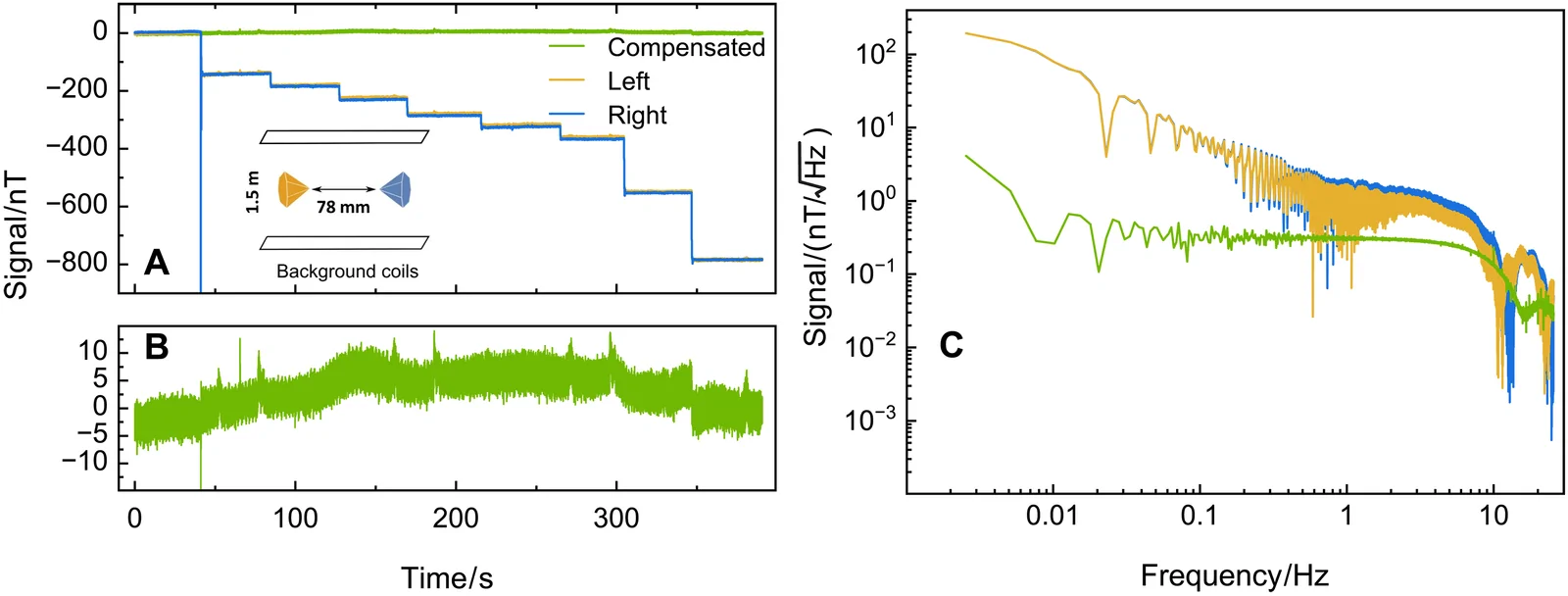
Characterization measurements of a gradiometry protocol using diamond sensors. (**A**) Time-domain traces from the two diamond sensors of ZAQuant used to characterize gradiometry performance (“right” sensor in blue and “left” sensor in orange), together with their differential signal (green), recorded under different background coil magnetic fields. Inset: Schematic illustrating the two diamond sensors, used to assess the gradiometric performance. (**B**) Zoomed in differential signal time trace. (**C**) Amplitude spectral density corresponding to the three magnetic traces shown in (A).

At ZAQuant, two NV sensor heads separated by 78 mm were placed within background-field Helmholtz coils (≈1.5 m in diameter) inside the single-layer magnetically shielded room (Fig. 3A, inset). Controlled background fields were applied to simulate ambient interference, while localized signals were introduced to emulate biomagnetic sources.

Magnetic test fields on the order of several hundred nanotesla were applied, and the resulting time-domain traces from both sensors were recorded. As shown in Fig. 3A, a simple subtraction of the two sensor signals was sufficient to cancel the applied test field and to substantially reduce the overall noise. Only the compensated data of Fig. 3A of the two magnetometers are displayed in Fig. 3B. The performance of the gradiometer scheme can be seen in Fig. 3C. It shows the noise spectral density (derived from the data in Fig. 3A) of both sensors and of the subtracted signal. The noise density of the individual sensors was larger than 100 $\text{nT}/\sqrt{\text{Hz}}$ at low millihertz frequencies and was suppressed by almost two orders of magnitude in the gradiometry trace. The linear range of the gradiometer is ultimately limited by the magnetic resonance linewidth (ZAQuant sensor, ∼200 kHz), degrading gradiometric suppression beyond ∼±500 nT.

This range can be extended via active frequency tracking and closed-loop feedback, enabling real-time operation under field variations of up to several microtesla, relevant for dynamic environments present for example in a neurosurgery theater. Furthermore, the scalability of the diamond platform supports gradiometric baselines from millimeter to meter scale, allowing system-level optimization for specific biomedical tasks. The gradiometric protocol successfully mitigates noise for the ZAQuant NV magnetometer, yet it cannot yet match the superior sensitivity of the shielded single sensor used in Stuttgart, being still an order of magnitude away in its final noise floor. These results demonstrate a clear pathway for implementing diamond sensor MCG detection in realistic, noisy environments using a gradiometric configuration.

**Fig. 4.**
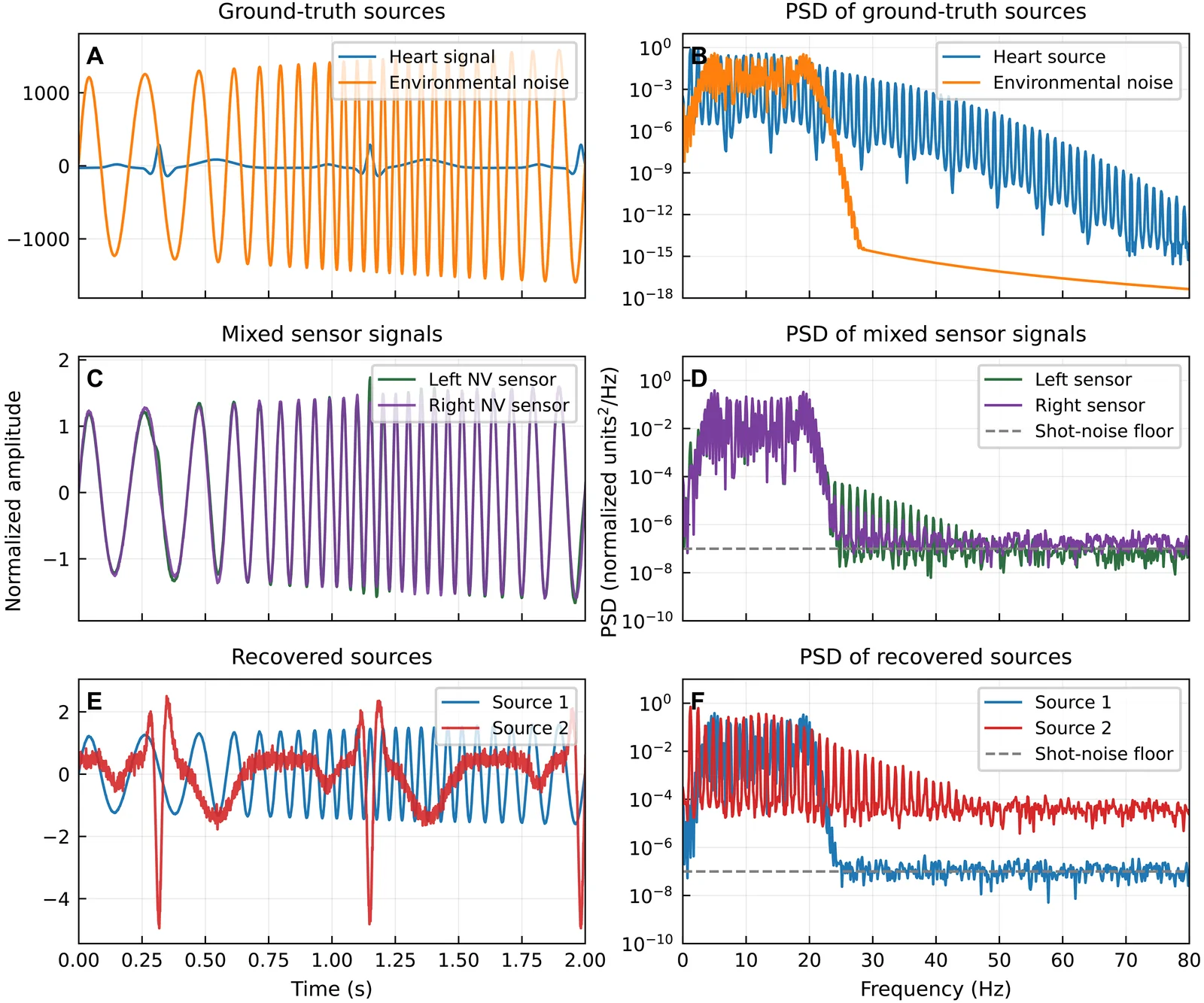
BSS of simulated two-channel NV-magnetometer recordings. (**A**) Time-domain ground-truth source signals, consisting of a cardiac-like magnetic signal and a stronger environmental magnetic noise component with time-varying frequency and amplitude. (**B**) Power spectral density (PSD) of the ground-truth sources. (**C**) Simulated mixed signals measured by two NV magnetometer channels, where the two channels contain different linear mixtures of the cardiac signal and the environmental noise. (**D**) PSDs of the mixed sensor signals, showing that the measured spectra are dominated by the environmental noise contribution. (**E**) Recovered source components obtained from BSS of the two mixed sensor signals, denoted as source 1 and source 2. (**F**) PSDs of the recovered source components. The dashed gray lines in (D) and (F) indicate the white-noise floor associated with the shot noise–limited sensitivity of the NV magnetometer. The time-domain traces in (C) and (E) are shown in normalized amplitude, and all PSDs are plotted in square normalized units per hertz.

Beyond the ideal common-mode case, however, direct subtraction is not always sufficient. In practical MCG measurements, environmental magnetic noise is rarely perfectly homogeneous across the gradiometric baseline. Small differences in sensor gain, orientation, local shielding response, and source geometry lead to unequal coupling of the same disturbance into the two channels. Moreover, interference in realistic laboratory or clinical environments can be nonstationary and may partially overlap with the cardiac spectrum, making simple frequency filtering or fixed-ratio subtraction insufficient. Under these conditions, the optimal gradiometric signal is generally not given by $x_{1}-x_{2}$ but by a data-adaptive linear combination of the sensor outputs. To illustrate this extended operating regime, we implemented a blind source separation (BSS)–assisted gradiometry analysis, as shown in Fig. 4. The simulation considers two NV magnetometer channels measuring different mixtures of a weak cardiac-like magnetic signal and a stronger environmental magnetic noise component with time-varying amplitude and frequency. The ground-truth sources are shown in Fig. 4 (A and B), while Fig. 4 (C and D) shows that the raw sensor channels are dominated by the environmental disturbance and do not directly reveal the cardiac component. Applying BSS to the two-channel data recovers two separated components corresponding to the cardiac-like signal and the environmental noise contribution, respectively Fig. 4 (E and F). Further details of the signal model, source separation algorithms are provided in the Supplementary Materials (*25*). This procedure does not require prior knowledge of the exact noise waveform but instead exploits the fact that cardiac and environmental sources couple differently to the two spatially separated sensors and exhibit distinct temporal or statistical structure. This analysis demonstrates that gradiometry can be generalized from a fixed differential measurement to an adaptive source separation problem. Whereas direct subtraction is most effective for spatially uniform noise with matched sensor responses, BSS-assisted gradiometry can compensate for unequal channel coupling and nonstationary interference. This provides a route toward more robust NV-based MCG operation in weakly shielded or dynamically changing environments, where the measured signals contain overlapping contributions from biomagnetic activity, environmental magnetic noise, and sensor noise.

## DISCUSSION

This work demonstrates human MCG with NV-based sensors, marking a crucial step toward clinical translation of quantum technologies. Table 1 situates this work within the broader context of MCG and ECG detection. While all three sensors exhibit comparable sensitivity, their SNRs vary substantially, reflecting the distinct magnetic environments in which they were operated. Multilayered magnetic shielding, as demonstrated by the Mainz sensor, enables longer averaging times by drastically reducing the magnetic noise floor. In unshielded environments, extensive filtering can partially compensate for limited averaging intervals, provided that the magnetic background remains sufficiently stable; this is observed in the Q.ANT sensor results. Although the Stuttgart sensor did not yield a convincing MCG detection with comparable SNR, exploring its gradiometric capabilities suggests a promising pathway for future mitigation. Currently, however, the gradiometric sensitivity is an order of magnitude lower than its single-sensor performance, necessitating further optimization of both sensors. The single-sensor characteristics are summarized in Table 2, and each sensor’s measurement modality is detailed in Materials and Methods. However, a substantial performance gap remains between current capabilities and the requirements for routine clinical or industrial use, primarily due to sensitivity limitations. The following question needs to be addressed to overcome these challenges: How can sensor sensitivity be increased to allow real-time detection with diagnostically sufficient SNRs?

**Table 2.** Overview of the three diamond sensors used in this manuscript and their characteristics.

Property	JGU	ZAQuant	Q.ANT
Diamond dimension (w × l × h)	0.5 mm by 0.5 mm by 0.18 mm	0.5 mm by 0.5 mm by 0.5 mm	1 mm by 1 mm by 0.5 mm
Sensitivity	$13\;\text{pT}/\sqrt{\text{Hz}}$	$7\;\text{pT}/\sqrt{\text{Hz}}$	$26\;\text{pT}/\sqrt{\text{Hz}}$
Magnetic bias	Zero dc bias	1-mT bias	1-mT bias
Modulation	Magnetic mod.	MW amplitude mod.	MW amplitude mod.
Operating location	Multilayer shielded	Single-layer shielded	Unshielded
Integration level	Handheld device with a cubic m sized rack	Table top setup	Fully miniaturized as handheld device
Optical power on the diamond	∼300 mW	∼300 mW	100 mW

**Fig. 5.**
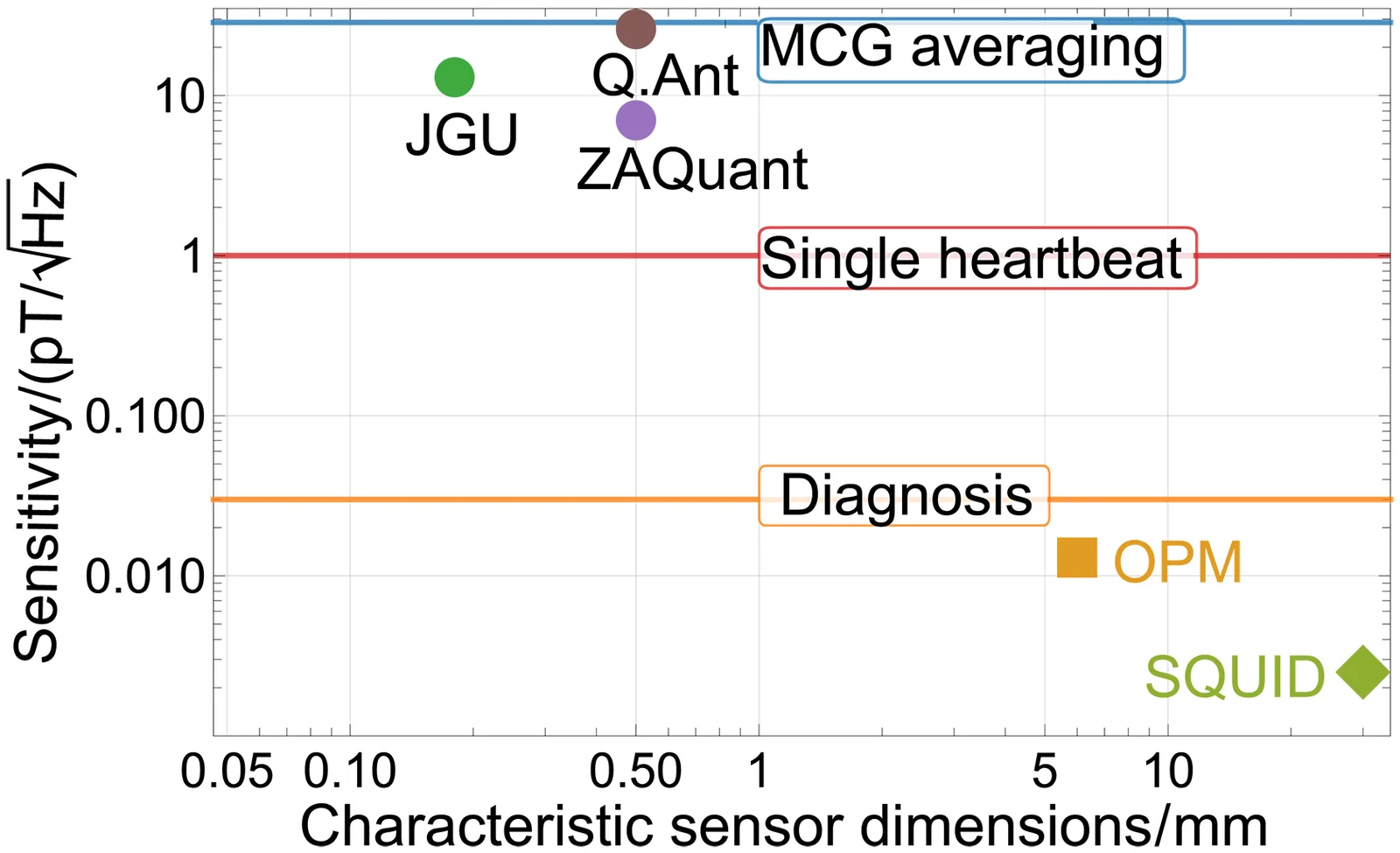
Comparison of NV based sensors for MCG detection. Including the present work, characterized by their sensitivity and a representative linear dimension of the sensing volume. Green, JGU; purple, ZAQuant; brown, Q.ANT; light green, SQUID (*47*); orange, OPM (*33*).

Recent NV-based magnetometry systems have achieved sub-10 $\text{pT}/\sqrt{\text{Hz}}$ sensitivities (*13*), positioning them as promising candidates within the broader magnetometry landscape. However, for clinical applications such as MCG and magnetoencephalography, sensitivities in the range of subpicotesla per square root of hertz—currently reached with SQUIDs and OPMs—remain necessary (*5*, *33*). An illustration of the achieved sensitivities within this work and estimated sensitivity targets for clinical relevance is displayed along with different sensing modalities and their characteristic sensor dimensions in Fig. 5. The performance of NV-based sensors allows the observation of averaged MCG, but clinical relevance requires larger signal-to-noise (*34*).

The baseline for live detection in Fig. 5 is determined by the measured signal amplitudes. Sensitivities on the order of 10 $\text{pT}/\sqrt{\text{Hz}}$ allow for the detection of MCG signals after sufficient averaging; however, reducing the required averaging time and enabling robust low-average or real-time MCG measurements will require further improvements toward ∼1 $\text{pT}/\sqrt{\text{Hz}}$. This corresponds to roughly an order-of-magnitude improvement over the sensors used in this work, but this performance has already been achieved in other NV center setups (*13*). Bridging this gap necessitates identifying and addressing the dominant limitations of current NV implementations. The NV ensemble magnetometers discussed in this work operate using continuous-wave protocols, which are approaching their practical sensitivity limits (*15*). In these systems, a dominant noise contribution arises from photon shot noise, scaling as $1/\sqrt{P}$, with *P* being the detected fluorescence power. While increasing optical excitation can reduce this noise, further improvements are constrained by power broadening, sample heating, and laser-induced decoherence.

To surpass this limit, several quantum-enhanced sensing strategies have been proposed and experimentally explored. Infrared absorption magnetometry offers a pathway for high-photon collection by spectroscopically interrogating the singlet transition of the NV center in diamond via the absorption of an infrared laser, potentially lowering the photon shot noise–limited sensitivity further (*35*, *36*). Pulsed magnetometry schemes exploit NV spin coherence more efficiently and suppress low-frequency technical noise via dynamical decoupling sequences, with demonstrated sensitivities reaching 490 fT/$\sqrt{\text{Hz}}$ (*13*). MW cavity–enhanced detection, by coupling NV ensembles to MW resonators under strong coupling conditions, may enable an order-of-magnitude sensitivity improvement (*37*, *38*). Many-body quantum effects, including spin squeezing and quantum gain amplification (*39*, *40*), offer potential routes to surpass the standard quantum limit, although their practical implementation remains at an early stage.

While quantum-enhanced strategies hold promise for long-term sensitivity improvements, their experimental complexity currently limits their near-term applicability. Magnetic flux concentrators offer a practical alternative for immediate performance enhancement (*16*, *41*). By channeling magnetic flux from a larger volume into the sensing region, these passive structures can amplify the local field experienced by the NV ensemble, yielding more than two orders of magnitude signal enhancement without requiring changes to the underlying measurement protocol (*16*, *18*). In addition to their technical simplicity, flux concentrators can be matched to many biomedical applications. An associated reduction in spatial resolution—due to flux collection over larger areas—can be acceptable in several biomagnetic sensing scenarios such as cardiac monitoring, where centimeter-scale localization is sufficient.

## MATERIALS AND METHODS

In this work, we developed three NV-diamond-based magnetometers. At JGU, a zero-field magnetometry protocol was used, achieving sensitivities comparable to bias field optically detected magnetic resonance (ODMR) spectroscopy (*42*). The zero-background compatibility enabled a direct comparison between diamond- and OPM-based MCG detection. The protocol relied on ODMR spectroscopy at zero magnetic bias field. A modulated magnetic field (6.12 kHz, microtesla amplitude) was applied along the [100] crystallographic direction of the diamond, allowing for the retrieval of a magnetically sensitive signal from NV centers along all crystal axes without requiring a static bias field (*20*, *21*, *43*).

The central MW frequency at 2.87 GHz was mixed with a 2.16-MHz radio frequency signal to address all hyperfine levels, enhancing resonance contrast and improving sensitivity. The central MW frequency was modulated (modulation frequency, 140 kHz; modulation amplitude, 100 kHz) and locked on resonance using the demodulated dispersive signal as the error signal in a feedback loop. The MW signal was delivered to the sensing diamond using a custom made printed circuit board. At zero bias field, this MW frequency locking renders the magnetically sensitive signal largely insensitive to temperature variations, enhancing the stability of long-term measurements.

The design of the sensing diamond and the diamond lens, optimized for photoluminescence collection, has been described in detail in (*23*). The sensing diamond and lens are mounted on a diamond plate for thermal management, which is, in turn, mounted on an aluminum housing (sensor head diameter, 12 mm). The housing integrates optics for laser delivery from a high-power fiber, collection of photoluminescence through a dichroic mirror, and detection via a differential photodiode board. The differential detection scheme minimizes the influence of laser noise on the signal. A customized holder for the optical fiber output coupler is also integrated into the sensor head. The sensor design is described in further detail in (*21*).

Fiber and signal cables are routed to a rack housing a breadboard, which supports a Coherent Verdi G5 laser head with integrated optics, a Zurich Instruments lock-in amplifier for data processing, associated MW electronics, and a personal computer (PC) for instrumentation control and data analysis. The Q.ANT sensor used a similar sensor head architecture, maintaining a compact overall footprint with a sensor head volume of 1 dm^3^. A fiber-coupled OBIS laser from Coherent provided 100 mW of light power at the fiber output and was used to pump the sensing diamond. The sensor system integrates a field-programmable gate array for control, MW generation, and a diamond (1 mm by 1 mm by 500 μm) with a truncated pyramid geometry containing ∼1.7 parts per million of NV^−^, as determined by electron paramagnetic resonance spectroscopy.

Fluorescence from the diamond was acquired using a low-noise balanced photodiode board with a differential amplifier, which simultaneously monitored part of the laser light to suppress intensity fluctuations, similar to the approach used at JGU. To resolve all four NV-resonance pairs corresponding to the four NV orientations in the diamond lattice, a bias field of ∼1 mT was applied via an in-house fabricated Halbach ring.

The sensor interfaces with a control PC via universal serial bus connection, running software that includes a graphical user interface for sensor control and real-time data visualization. A sophisticated miniaturized optical design collected diamond fluorescence with an estimated efficiency of roughly 40%, as determined from optical simulations.

At ZAQuant, the diamond sensors were similarly designed as the Q.ANT sensors. As shown in Fig. 1D, the optical module consisted of a green laser excitation path and a compact fluorescence detection stack with dimensions of ∼12 mm by 12 mm by 95 mm. Note that this does not include the MW and bias field delivery system. For the gradiometric operations, two identical sensor heads were arranged in a mirrored geometry, such that the sensors face each other. This allowed precise alignment of the magnetometers. The electronics subsystem included MW signal generation and signal readout with a Zurich Instruments HF2LI lock-in amplifier, all placed outside of the shielded measurement room to reduce electronic noise. A double-resonance scheme combined with hyperfine driving was implemented to address all three hyperfine transitions of a selected NV axis similar to the JGU sensor. A 3-kHz MW frequency modulation was applied to enable lock-in detection with enhanced sensitivity (*25*). The chosen modulation frequency balances signal contrast and noise suppression (*44*). The same architecture was duplicated for the second spin transition with opposite modulation phase, and the resulting MW signals were amplified and delivered to the diamond via a single loop antenna.

### Ethics statements

No ethics approval was required for the noninvasive self-experiments (personal communication of the local Ethics Committee of the University of Freiburg to T.B.).

## Supplementary Materials

### This PDF file includes:

Sections S1 to S4 Figs. S1 to S17 Table S1 References
