# Supplementary material for: Human cardiac measurements with diamond magnetometers
Source: Sci Adv. 2026 Sep 16;12(38):eaeg5281. doi: 10.1126/sciadv.aeg5281 (PMC13580569; doi:10.1126/sciadv.aeg5281)
Supplement: Supplementary file 1 [file sciadv.aeg5281_sm.pdf]

Science Advances
AAAS

Supplementary Materials for

Human cardiac measurements with diamond magnetometers

Muhib Omar *et al.*

Corresponding author: Shaowen Zhang, shzhang@uni-mainz.de; Jixing Zhang, jixing.zhang@pi3.uni-stuttgart.de

Sci. Adv. **12**, eaeg5281 (2026)
DOI: 10.1126/sciadv.aeg5281

This PDF file includes:

Sections S1 to S4
Figs. S1 to S17
Table S1
References

A Details of the Mainz NV Magnetometer

A.1 Sensor Design and Assembly

The magnetometer was designed with a compact 12 mm diameter footprint to facilitate versatile integration. The resulting sensor head, housed within a magnetic shield, is shown in Fig. S3, with core components detailed in Fig. S1. The diamond-based collection optics are described in the main text's methods section with the corresponding reference.
The microwave (MW) delivery and photodiode printed circuit board (PCB) designs are illustrated in Fig. S2. A flexible PCB ($\approx$ 150 μm thick) is used for MW delivery; it features a central aperture through which the sensing diamond protrudes by approximately 100 μm. This geometry was specifically chosen to allow for the application of circularly polarized microwaves.
The photodiode PCB was engineered for high compactness and the elimination of magnetically active components. We utilized non-magnetic photodiodes (Hamamatsu S13228-01) and secured all aluminum housing components with titanium screws (TI AL GmbH) to minimize residual magnetic signatures.
The sensing assembly consists of a [100]-cut "science" diamond [Element Six (UK) Ltd.] mounted to a diamond anvil (Dutch Diamond Technologies BV) using a high-refractive-index optical adhesive (Norland Optical Adhesive 170). This anvil is fixated to a polycrystalline diamond plate (custom-fabricated by Medidia Tec GmbH), which is then screwed to an aluminum cylinder. To ensure efficient thermal management, a silicate-based thermal paste was applied between the diamond plate and the cylinder. This configuration maintains the diamond tip temperature below 40°C during operation, theoretically allowing for direct contact measurements of biomagnetic signatures from skin or biological tissue.

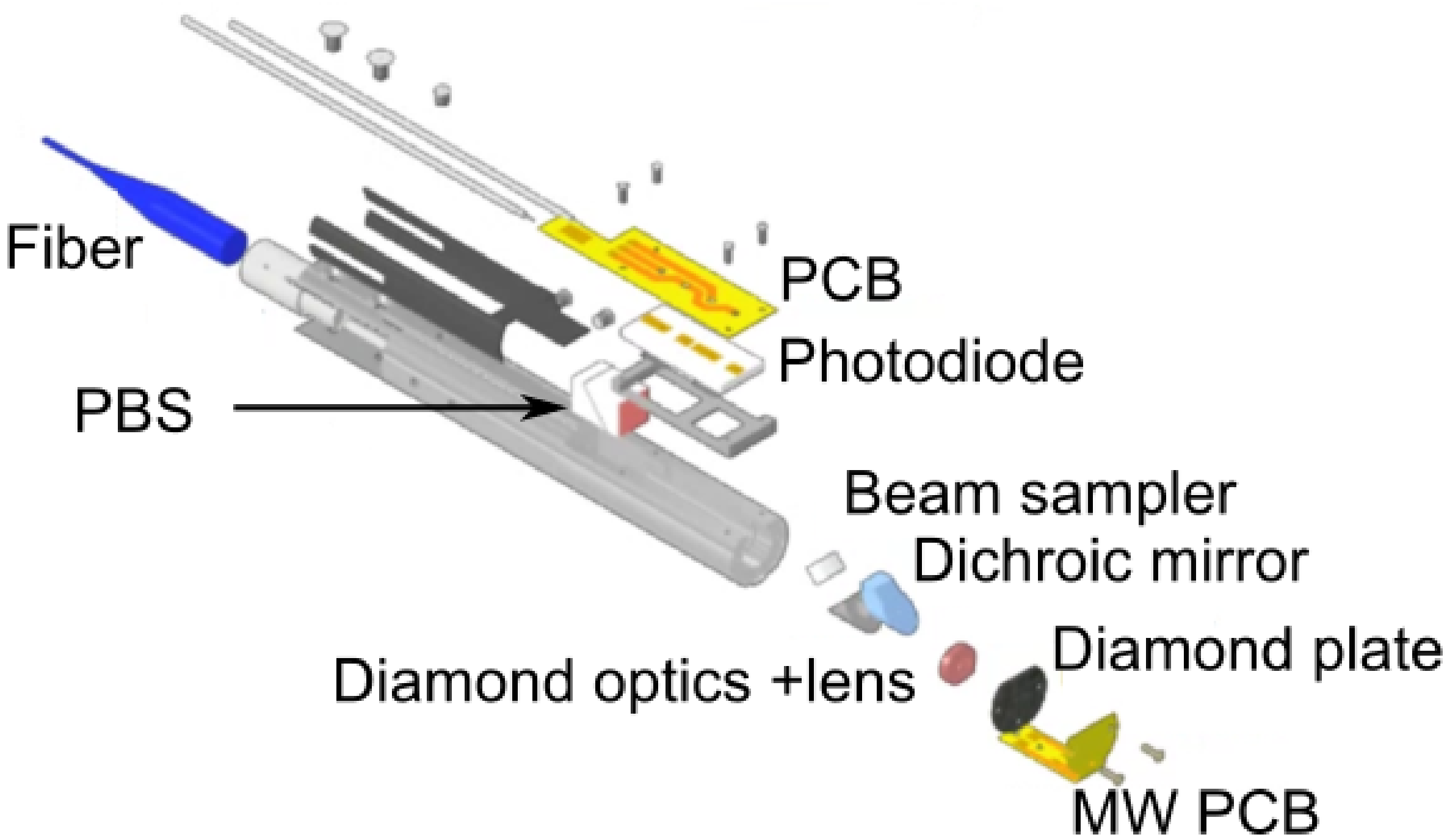

Fig. S1: The Mainz endoscope sensor head component view.

A.2 Sensing Principles and Zero-Field Linearization

The system utilizes zero-field optically detected magnetic resonance (ODMR) spectroscopy. A MW driving field induces transitions between electron spin states, which are detected as variations in photoluminescence (PL). At resonance, the transition from a "bright" to a "dark" spin

state creates a Lorentzian resonance dip. While the Zeeman effect provides linear frequency shifts in high bias fields, the response at zero field is inherently quadratic. This occurs because multiple spin transitions overlap at zero field; an external magnetic field causes these transitions to move in opposite directions, resulting in an overall broadening of the resonance line rather than a directional shift.

To achieve linear sensitivity, we apply an oscillating RF magnetic field via a five-turn coil wound around the aluminum cylinder tip. This modulation allows us to extract a dispersive signal from the resonance dip depth. The modulation coil was calibrated using high-field ODMR to map the magnetic field response to the lock-in amplifier (LIA) input voltage (see Fig. S4 and Fig. S5).

A.3 Optimization and Laser Noise Suppression

The sensitivity of the magnetometer is determined by the slope of the dispersive signal. To optimize this, we apply a magnetic test signal and monitor its magnitude (the R-component of a second demodulation, hereafter "Demod R2"). By sweeping the MW frequency, we identify two specific frequencies in the zero-field ODMR spectrum where the magnetic response is maximized (Fig. S6). Driving both frequencies simultaneously with a dual-frequency MW source further enhances sensitivity. Following frequency identification, the MW power for each frequency is optimized by maximizing the Demod R2 signal (Fig. S7 and S8).

Finally, common-mode laser noise is suppressed using a balanced differential photodiode circuit. The performance of this circuit depends on the precise balancing of light intensity between the two photodiodes. We characterized the system's ability to reject laser modulation (simulating noise) as a function of photodiode imbalance (Fig. S9). Currently, balancing is achieved by manually adjusting an optical filter to attenuate the reference green laser light; however, an automated electronic compensation system is under development.

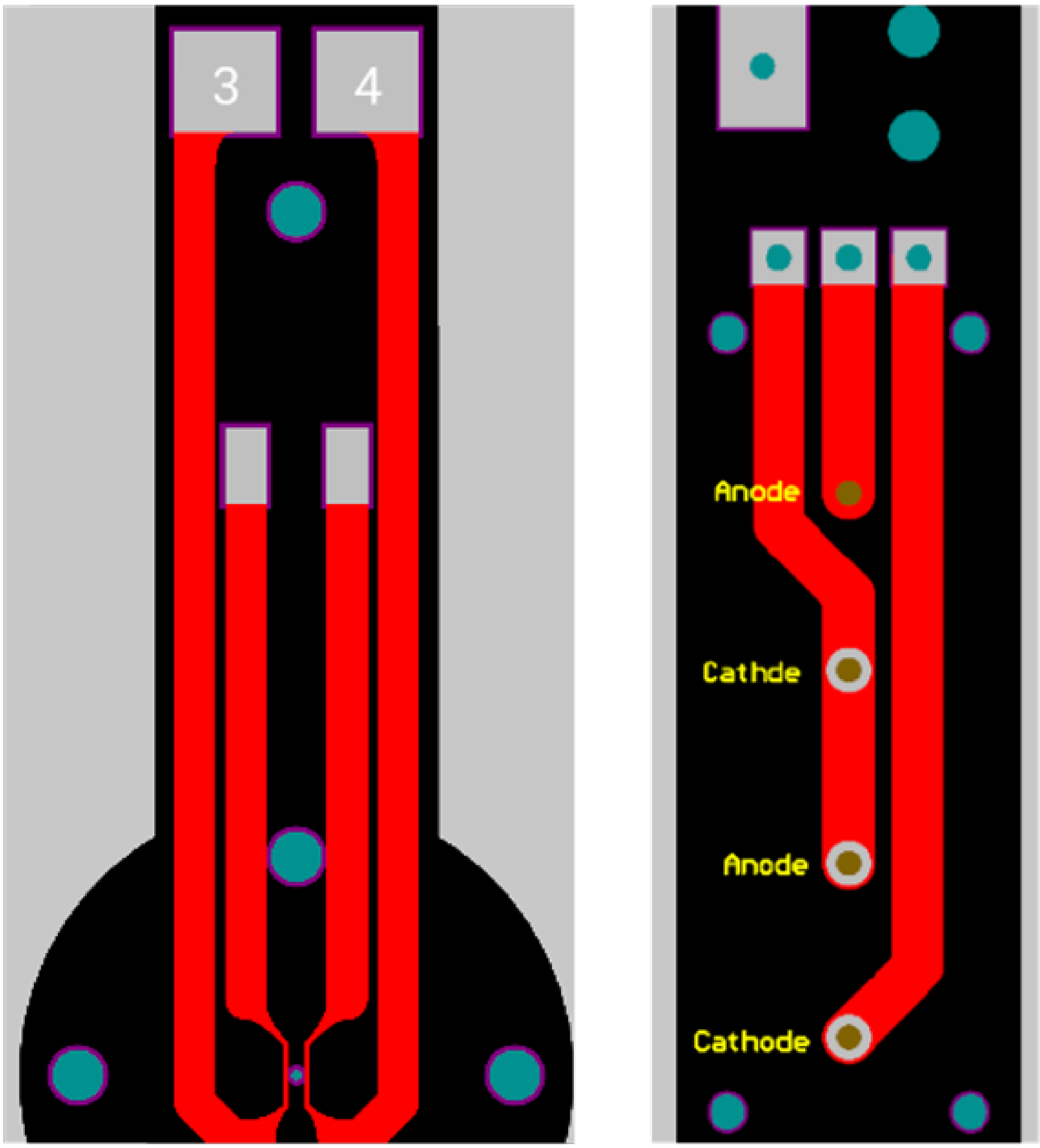

Fig. S2: The microwave delivery PCB (left) and the photodiode assembly PCB (right).

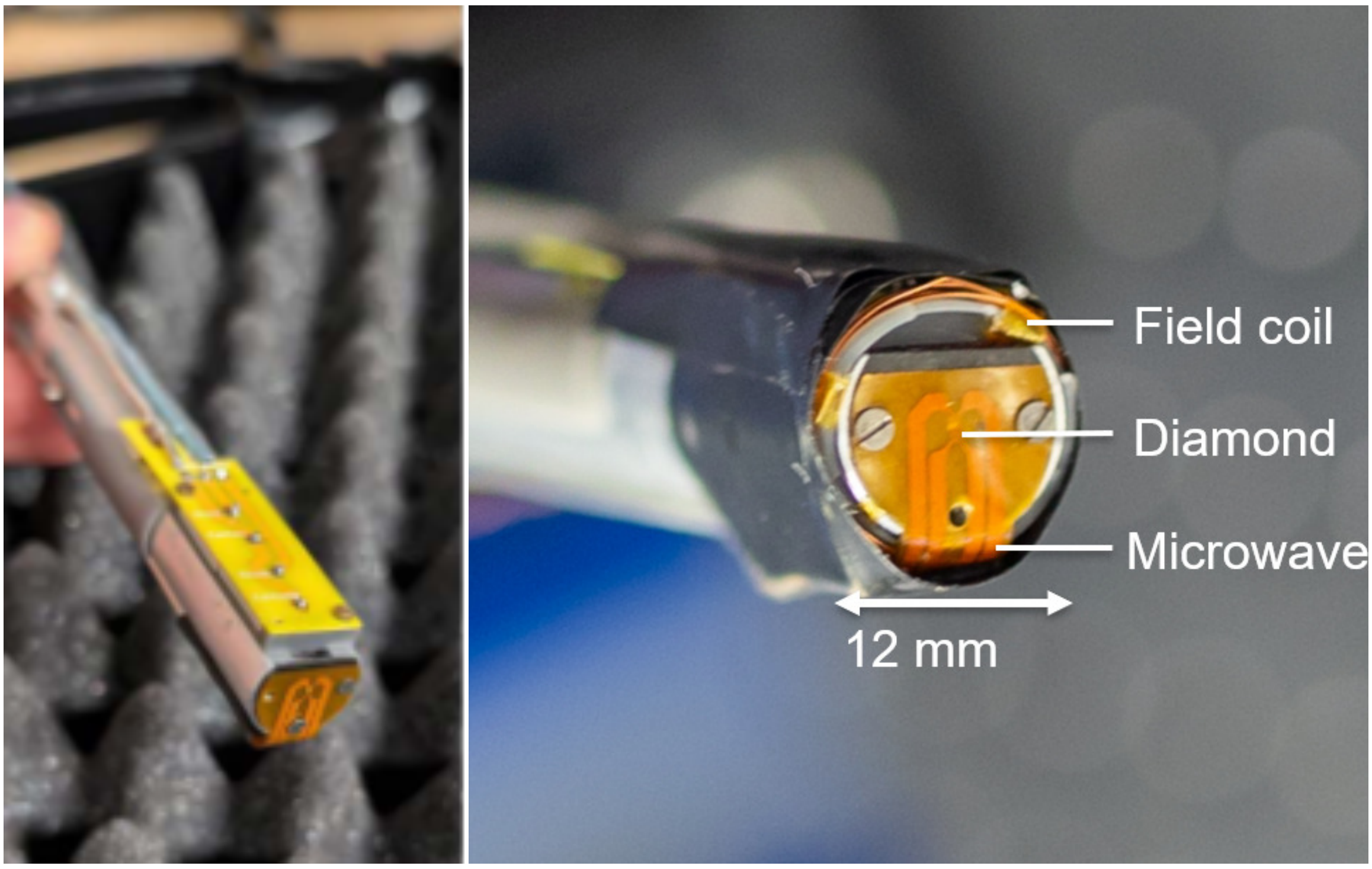

Fig. S3: Photographs of the sensor head (left), annotated close-up of the sensor head(right).

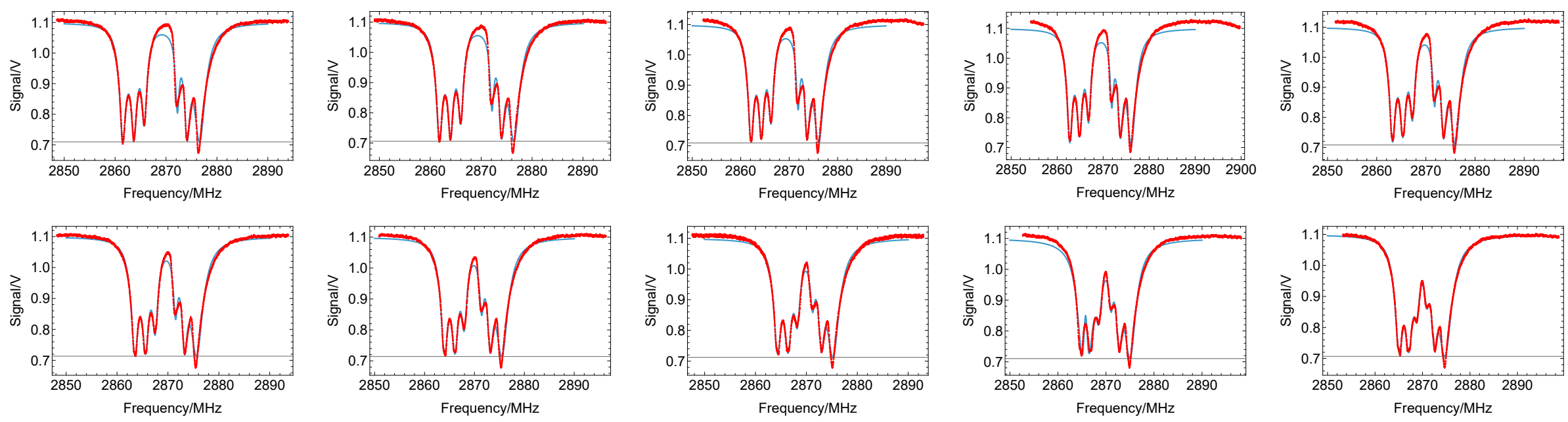

Fig. S4: High-field ODMR spectra for varying voltages applied to the modulation coil (ranging from $-10\,\mathbf{V}$ to $+10\,\mathbf{V}$).

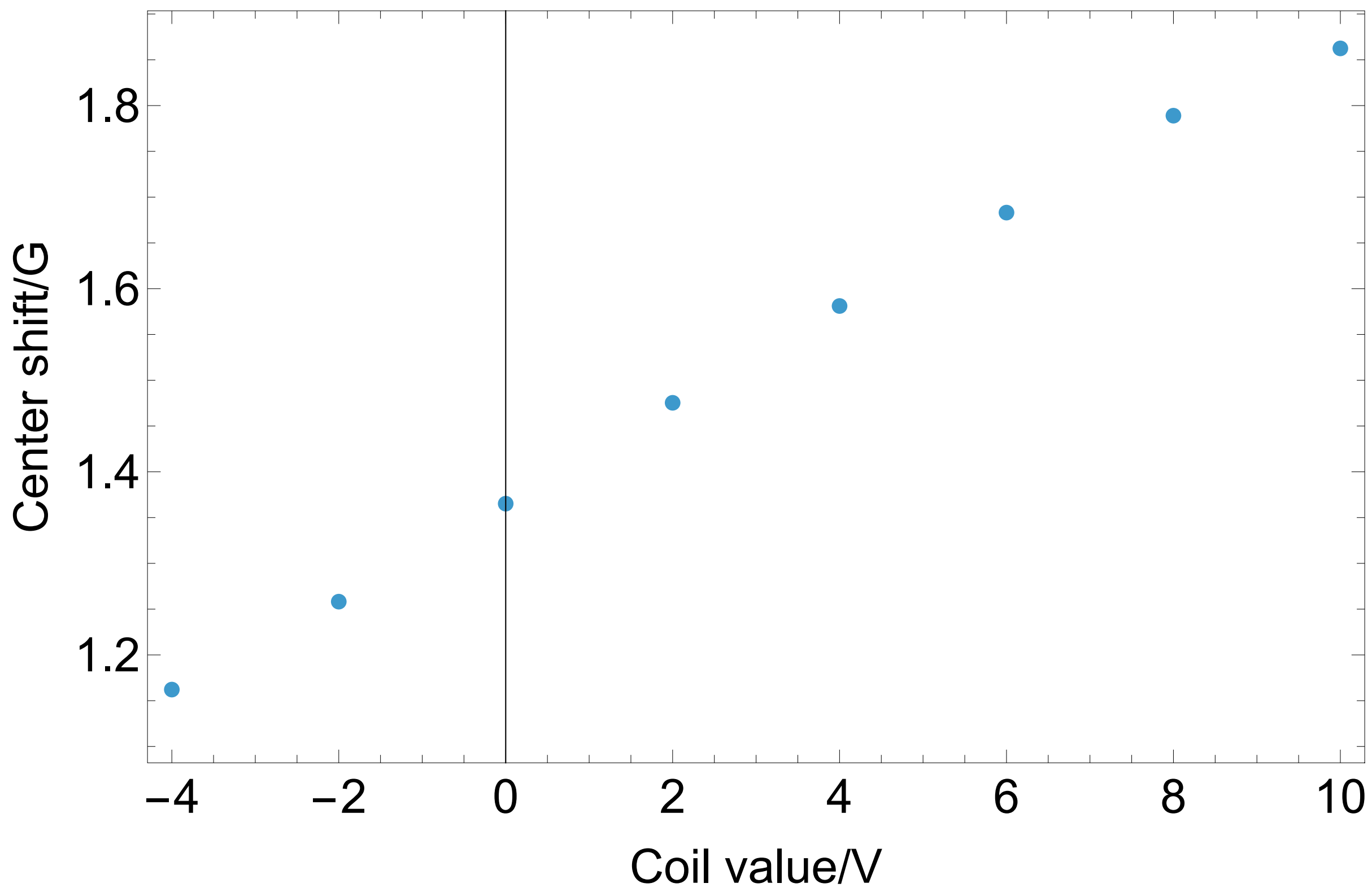

Fig. S5: Extracted magnetic field as a function of the applied LIA voltage, derived from the ODMR spectra.

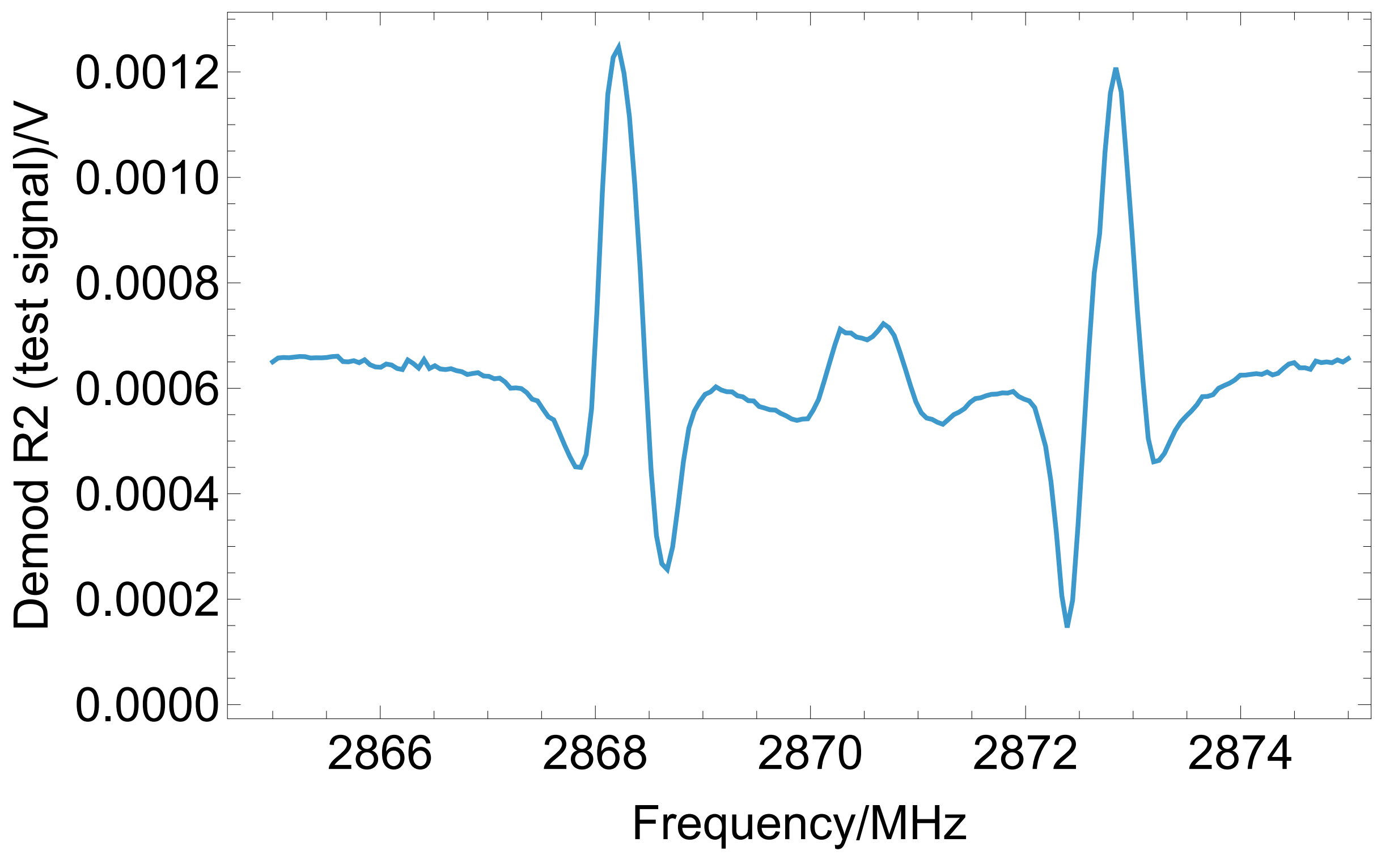

Fig. S6: Magnetic test signal magnitude (Demod R2) as a function of MW frequency, identifying the points of maximal magnetic response.

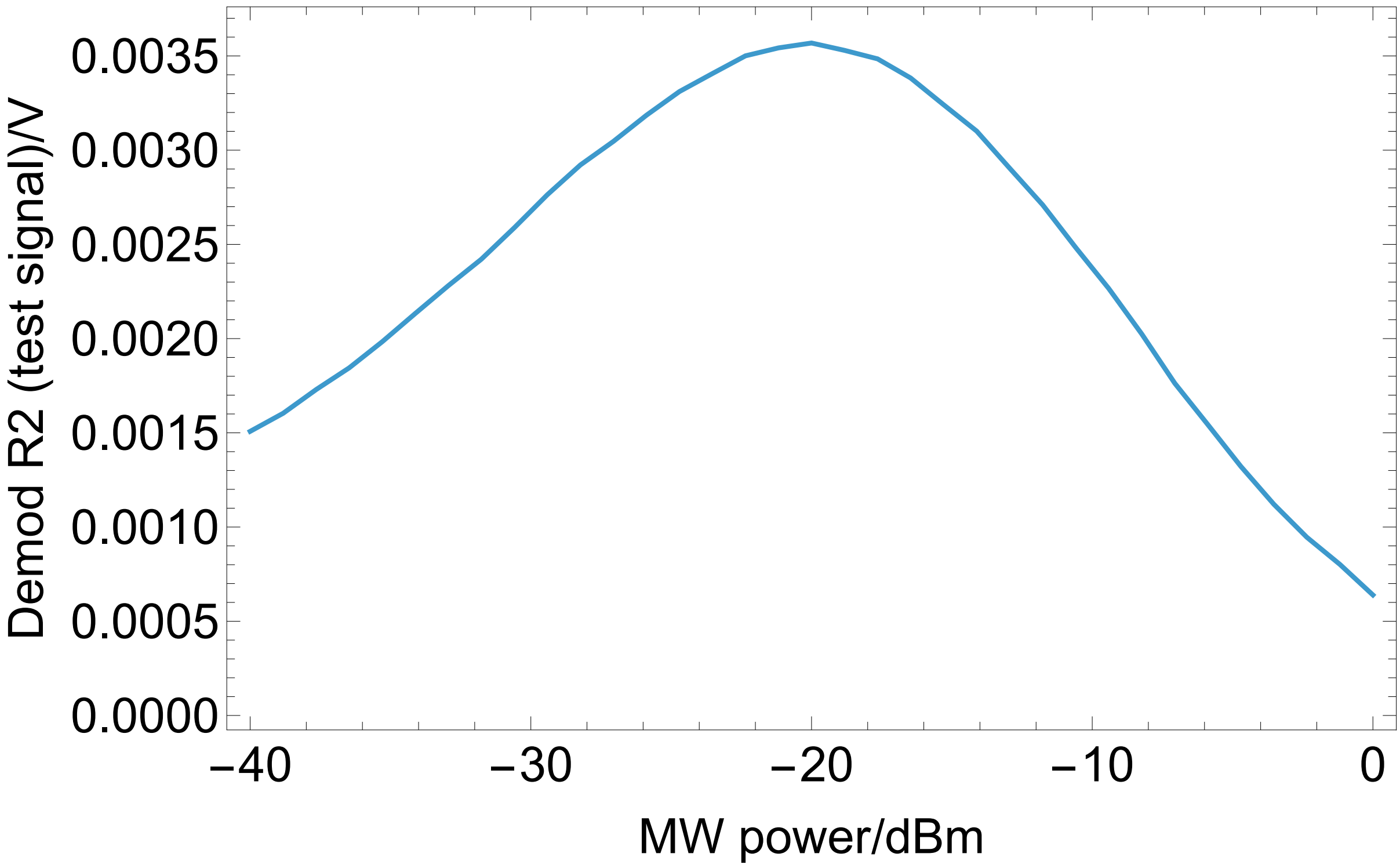

Fig. S7: Optimization of MW power for the first identified resonance frequency.

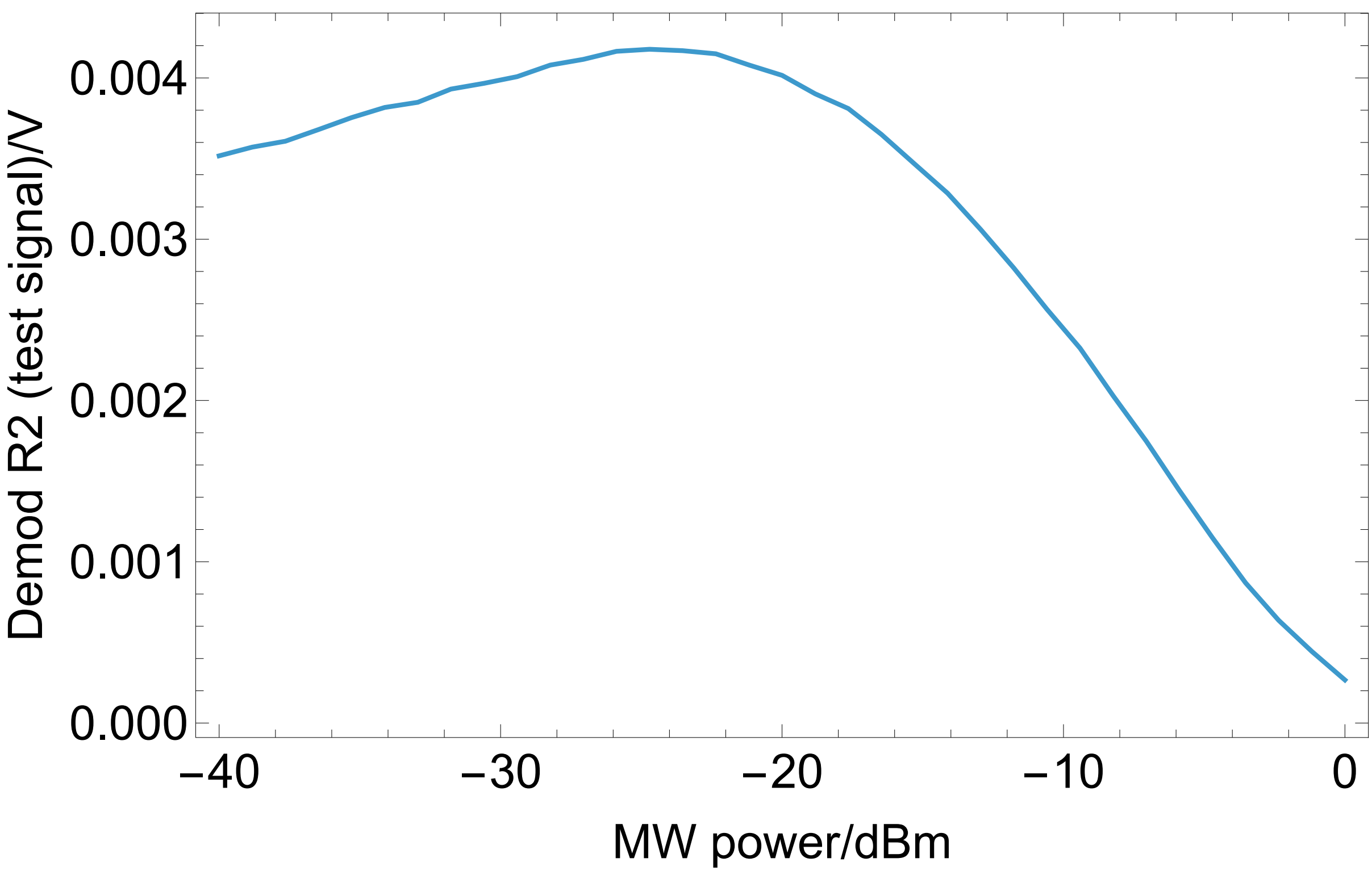

Fig. S8: Optimization of MW power for the second identified resonance frequency.

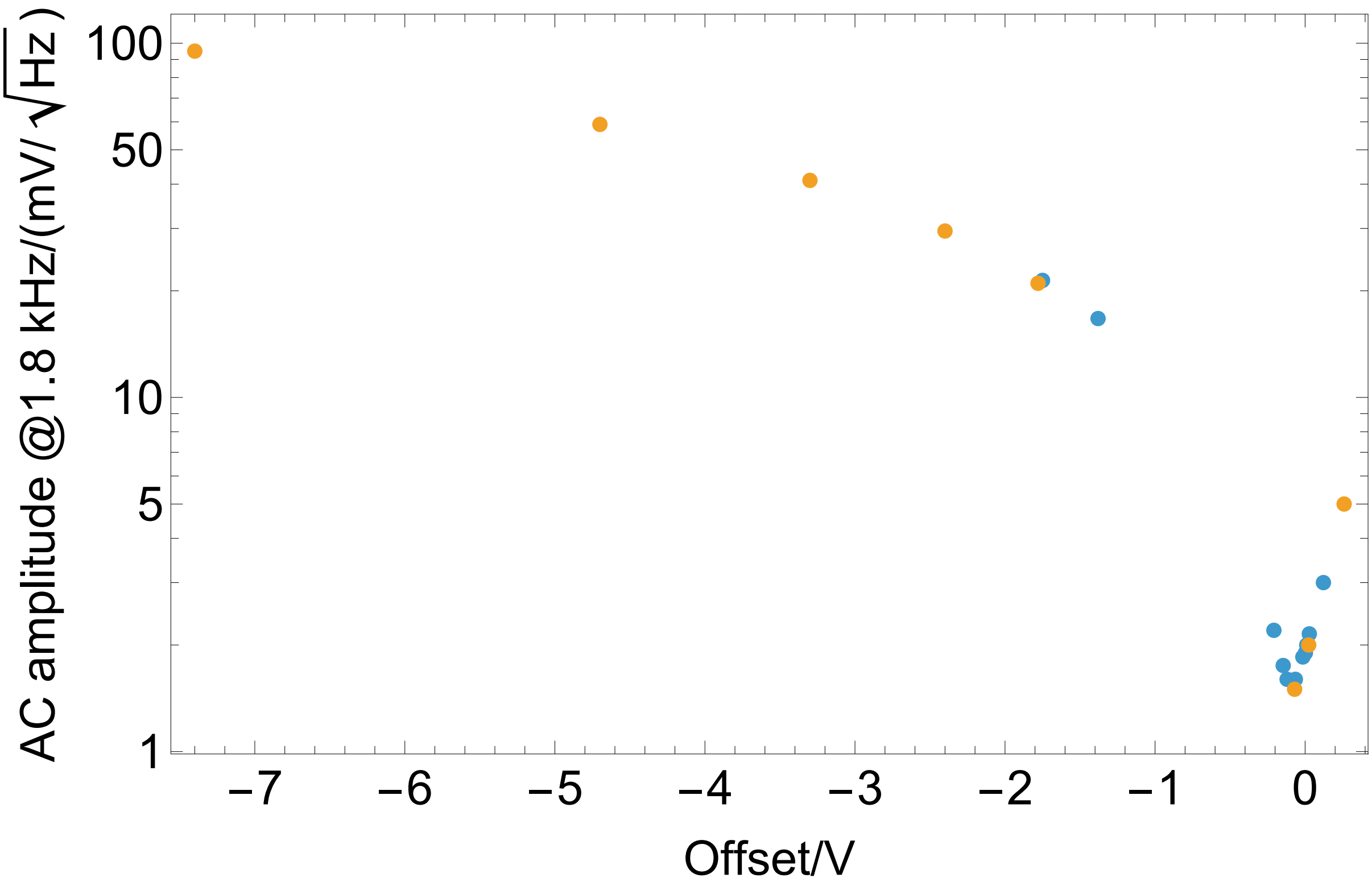

Fig. S9: Suppression of laser noise as a function of photodiode circuit balancing.

B Details for Stuttgart NV magnetometer

The sensitivity of the magnetometer directly improves with a higher number of detected photons. Therefore, the effort to increase the photon number, mainly by improving the collection efficiency of the fluorescence, started together with the proposal of the NV center as magnetometer. This includes both solutions for single NVs (*48-52*) as well as for ensembles of NV (*23, 53-55*).
In ensemble sensing, where the size of the diamond is much larger than the size of the NV center, the main focus is on the optimization of the geometry of the diamond and the collection optics. Five key features the optical sensor part should fulfill can be identified:

1. High collection efficiency
2. Low interference between electronics and NV
3. Easy and safe usage
4. Optimized detection volume
5. High excitation efficiency

Here we sort the features by their importance for our application-related focus in descending order. The most important factor is the total collection efficiency in order to reach the best sensitivity. We here define it as the power measured at the photo-detector divided by the total generated fluorescence power.
Secondly, for a high sensitivity sensor currents from any surrounding electronics will contribute to the magnetic background noise. Most devices can be placed far away from the sensor head, however, the PD (Photodiode) needs to be close to the diamond to preserve the high collection efficiency from the first key feature. This means an optical design is necessary to separate the two components without the loss of fluorescence power.
In a real application scenario a biosensor needs to be safe for everyone involved, meaning no laser light and microwave fields should be able to reach the test object or person as well as the operators. An easy usage means that the measurement time should be minimized such that a good SNR is achieved and the averaging time to discern the signal is short. This is achieved by placing the sensor head in close proximity to the target so the signal strength is larger, naturally improving the detection limit. Additionally, placing the sensor close to the target increases the spatial resolution of the measurement to identify the origin of a signal more precisely.
The detection volume, i.e. the diamond size, directly limits the spatial resolution. On the contrary if the NV-concentration is fixed while the volume is reduced the fluorescence is decreased and the sensitivity reduced. This means in a real application there needs to be a compromise between spatial resolution and sensitivity.
Lastly, by increasing the excitation efficiency the necessary laser power is reduced. This not only decreases the power consumption of the whole sensor but also reduces the size of the heat dissipation system resulting in a smaller sensor. To incorporate the laser excitation into the overall collection efficiency we define a new parameter, the conversion efficiency. This parameter defines the ratio between the detected fluorescence at the PD and the excitation laser power and can be used as benchmark parameter for the quality of the optical design.

By keeping these features in mind the design of our sensor is as follows:
The components of the optical stack consist of the diamond (Element Six (UK) Ltd.), a CPC (Compound Parabolic Concentrator, Edmund Optics #65-443), a glass rod, a dichroic cube (both Union Optics) and a filter (Newport 20CGA-695, cutted in 10 mm × 10 mm shapes) as depicted in the main text. The green laser light enters the optics from the back through the dichroic beamsplitter and is transmitted to the glass rod. The glass rod acts as light pipe and guides the laser beam further. Because of its length (75 mm) it separates the diamond and

the PD, which reduces the magnetic background field from the electronics. Losses along the rod are minimal and can be ignored. On the second side of the rod the CPC lens with the larger diameter (5.39 mm) is attached, using the properties of the CPC as efficient light collector *(44)*. The diamond is then placed on the small side of the CPC-lens (diameter 2.5 mm). The generated fluorescence will take the same path back to the dichroic cube where it is reflected through a 695 nm cut-on wavelength filter to the photodiode (Hamamatsu S3590-09). The filter blocks any residual laser light as well as enhances the NV^- to NV^0 ratio, which improves the obtained contrast. To further increase the contrast of the measured single NV-axis a $\lambda/2$-plate is used to optimize the polarization of the laser excitation. For a diamond with (111)-cutting this can increase the contrast of the ODMR spectrum by up to 50 percentage points (Fig. S10b left). For a (100)-cutting, as used in our setup, the improvement is not as significant due to the angle of the NV-axis of interest but still 30 percentage points larger than under unfavorable polarization (Fig. S10b right). Because the linewidth does not follow this behavior the contrast increase directly translates into an improvement of the sensitivity. The microwave and magnetic bias field are delivered by generic commercially available loop antenna and a permanent magnet respectively. The antenna has a loop diameter of 10 mm and the ring magnet a size of 70 mm × 32 mm × 15 mm. The magnet is aligned to the (100)-axis of the diamond.

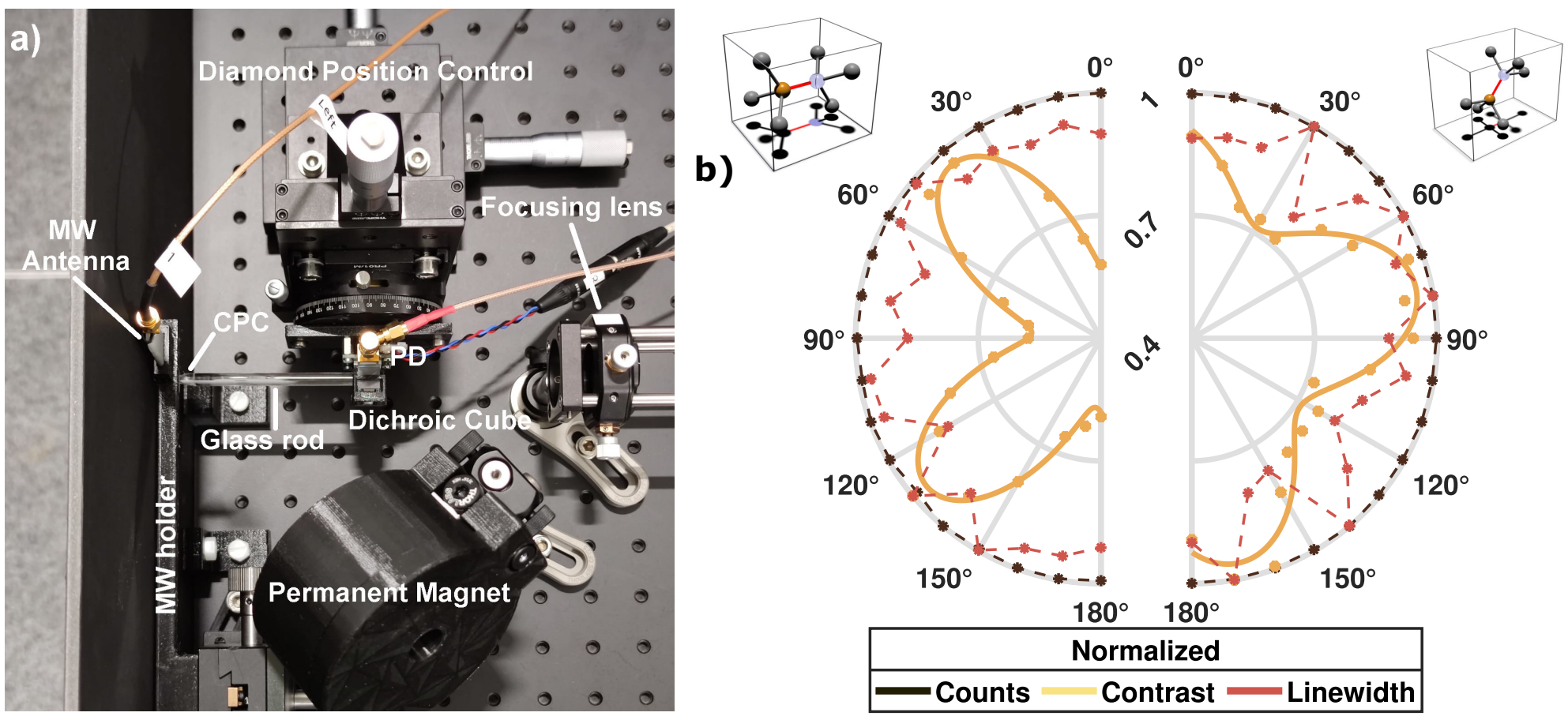

Fig. S10: MCG measurement setup and polarization-dependent ODMR performance. (a) Image of the MCG measurement setup. The diamond is hidden inside of the antenna on top of the CPC. The reference path and polarization control are outside of the image. For a full description refer to the text. (b) Polarization dependency of the ODMR contrast and linewidth for both a (111)-cut diamond (left) and a (100)-cut one (right). For all measurements the achieved count rate (black) was kept constant by adjusting the laser power entering the system. The contrast is fitted with a sinusoidal function, all other traces are only added as guide for the eye. The angle defines the angle between the polarizer fast axis and a vertical orientation.

B.1 Diamond shaping

The most sophisticated improvement in our sensor design is the shape of the diamond. For most works a cubic or rectangular shaped diamond is used. However, this comes with multiple caveats. Only the fluorescence exiting the diamond on one of six sides is collected. And because in a cubic shape closed paths which traps the fluorescence inside the diamond exist, the out-coupled light is further limited. Lastly in this geometry the overall excitation efficiency is low. To improve the collection two possible paths can be taken: Either new structures around

the diamond are added or the diamond itself is shaped into the desired form. Using collection structures around the diamond has the advantage that a different material, which is much easier to work with than diamond, can be used. To directly produce the shapes we chose a two-photon polymerization process (nano 3d-printing) and a specialized printing resin with a refractive index of 1.51 (micro resist technology OrmoComp®). This resin also showed a low auto-fluorescence, as discussed in the next section, which reduces its influence on the sensitivity. To further enhance the collection efficiency the structures are coated with silver. While the additional system around the diamond is a promising candidate, using only the diamond and shaping it directly is much simpler optically, as there are fewer components and therefore interfaces potentially increasing the fluorescence absorption. Because the materials for 3d printing are limited and most of them have a similar refractive index, the out-coupled light of the diamond is physically limited, which is given by the ratio of the refractive indices. Also the 3d-printing and coating will produce non-ideal surfaces, which will limit the improvement of the collection efficiency to reach the simulated optimum, as will be evident later. Therefore we simulated and tested the shaped diamond simultaneously.

The two most promising shapes are an aspheric lens completely covering the diamond and side-pyramids attached to the side of the diamond. For the direct diamond shaping only a simple pyramid was considered, as more complex shapes could not be fabricated. All simulated shapes are compared in Fig. S11a. The diamond cube on top of the CPC-lens, as used before and as benchmarking system, achieved an overall collected fluorescence (OCF), which is the percentage of fluorescence reaching the output of the glas-rod compared to the total produced fluorescence in the diamond, of 22 %. This confirms the loss of a major part of the fluorescence through single sided as well as limited out-coupling. The coated aspheric lens achieved a maximum OCF of 65 %, which approaches the physical limitation of the out-coupled light from the diamond with this kind of structure. However, the optimal OCF requires the diameter of the lens to be as large as the CPC lens, which is hard to fabricate. For more reasonable lens sizes the OCF is slightly reduced to around 60 %, which is still an improvement by a factor of 2.7 over the benchmark system. In the experiment the collected fluorescence improved by 2.7 (Fig. S11b left center) compared to the cubic diamond, which is much smaller than the expected enhancement of more than four times. This is most likely a problem of imperfections in the fabrication process and in the assembly of the optical system.
This is amplified by the size of the aspheric lens, even for the smallest one a multi-part print is required. While the lens is printed in one process, the working volume of the machine is not large enough to fit the whole lens. Therefore, the printing head needs to reset its position multiple times per print, introducing edge effects inside of the lens, degrading its optical properties. One solution to this is the use of side pyramids, which can be printed individually and will cover only the four sides of the cubic diamond. This promises an OCF of 55 % for a side length of 300 µm, with the outer side of the pyramids coated in silver. Larger sizes of the pyramid could further enhance the OCF, but were not feasible for printing. Note that by using side pyramids out of diamond one could theoretically improve the out-coupling even further to almost 80 %, however fabricating these shapes at the needed sizes proved to be difficult. The measured enhancement again falls short of the expected value and only reaches 1.9 (Fig. S11b center right). This is even lower than the aspheric lens even if the simulation initially suggested a better performance. The most likely explanation to this is the attachment process, which proved to be difficult to first remove the pyramids from the printing surface without damages and precise alignment around the diamond. For the lens one could simply place the diamond inside of the negative cutout in the lens, for the side-pyramids no such structure could be used to position them.
One additional problem with the coated shapes is the reduction of the driving microwave field. Simulations suggested that the used coating thickness of <50 nm should only marginally interfere with the microwave field and only reduce the strength by a few percent. However, when

experimentally tested we observed a decrease of the microwave strength by a factor of 1.3. The most likely explanation of the decrease is a combination of the silver reduction effect and inhomogeneities in the microwave field.

By directly shaping the diamond into the desired geometry no coating of the structure is necessary to achieve a similar OCF of 65 %. The optimal simulated shape is a truncated pyramid with a base length of 0.5 mm and an pyramid angle of 65°. Coating the pyramid diamond or embedding it into an aspheric lens, similar to the one discussed above, would further increase the collection efficiency to 82 % or 95 % respectively, but after the problems with the coated lenses we did not further pursue this option. The diamond used in the experiment had a slightly reduced angle of 63° and a small 10 µm truncation at the bottom because of fabrication limitations. Both restrictions are expected to reduced the achieved improvement only marginally. The detected fluorescence increased in the experiment by a factor of 8.2 compared to a cubic diamond, which is close to the expected improvement obtained by simulations (Fig. S11b right). In total the pyramid shaped diamond achieved an conversion efficiency of 2.7 % which is an improvement by a factor of 2.14 to the cubic diamond. If the losses due to the optics, as discussed in the next section, are factored in, the conversion efficiency can be increased to more than 3 %, providing a significant improvement to previous work where the conversion efficiency is typically below 2 %(*53, 56*).
For the final design in the sensor we therefore settled on the uncoated pyramidal shaped diamond, as this provided the largest improvement over the cubic diamond and no other drawbacks.

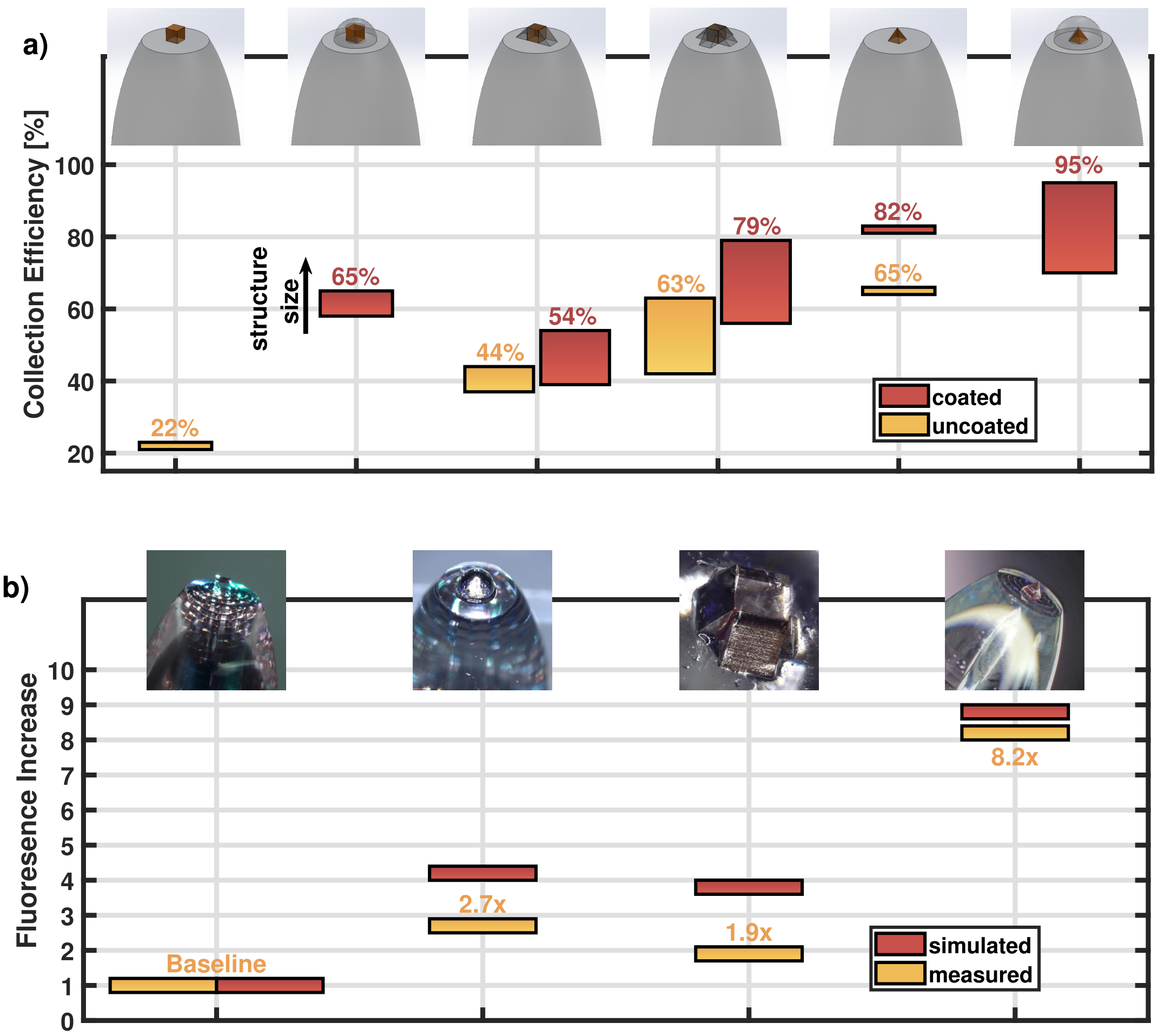

Fig. S11: Optimization of fluorescence collection from diamond sensor-head geometries. (a) Simulated collection efficiencies for different structures of and around the diamond on top of the CPC lens. From left to right: cubic diamond, aspheric lens, two and four side-pyramids, pyramid shaped diamond and pyramid shaped diamond together with an aspheric lens as depicted on the top. Generally a higher collection efficiency corresponds to a larger structure size. Numbers denote the highest achieved collection efficiency with the corresponding structure. (b) Measured increase of the collected fluorescence. From left to right: cubic diamond, aspheric lens, four side-pyramids and pyramid shaped diamond as depicted on the top. Differences between simulated and measured values are a result of imperfect fabrication methods. Numbers denote the measured increase compared to the baseline of the cubic diamond.

B.2 Sensor stack

All parts are glued together using an UV-cured adhesive to reduce air gaps and mounting points, which reduces power losses and alignment time. All used adhesives have at least two order of magnitude lower fluorescence over the whole NV spectrum compared to the NV itself when illuminated with the green excitation laser as shown in Fig. S12 (micro resist technology OrmoComp® (dark red) and OrmoClear® (light red) and M-Glas: Sigma-Aldrich Cat. No. 103973 (dark yellow)) This is important as any auto-fluorescence of the adhesive will reduce the contrast of the measurement and therefore the achieved sensitivity. One of the first tested adhesives (Nanoscribe IP-S, light pink curve) showed an auto-fluorescence almost comparable to the fluorescence of the diamond and we therefore disregarded it from further usage in the sensor.

Testing of the complete optical stack showed that 87 % of the laser light is transmitted to the diamond and 86 % of fluorescence reaches the filter. This means in total 25 % of the light is lost. This is one of the major bottlenecks of the system, by reducing these losses in the future much better conversion and collection efficiencies would be achievable, possibly improving the sensitivity to new records.

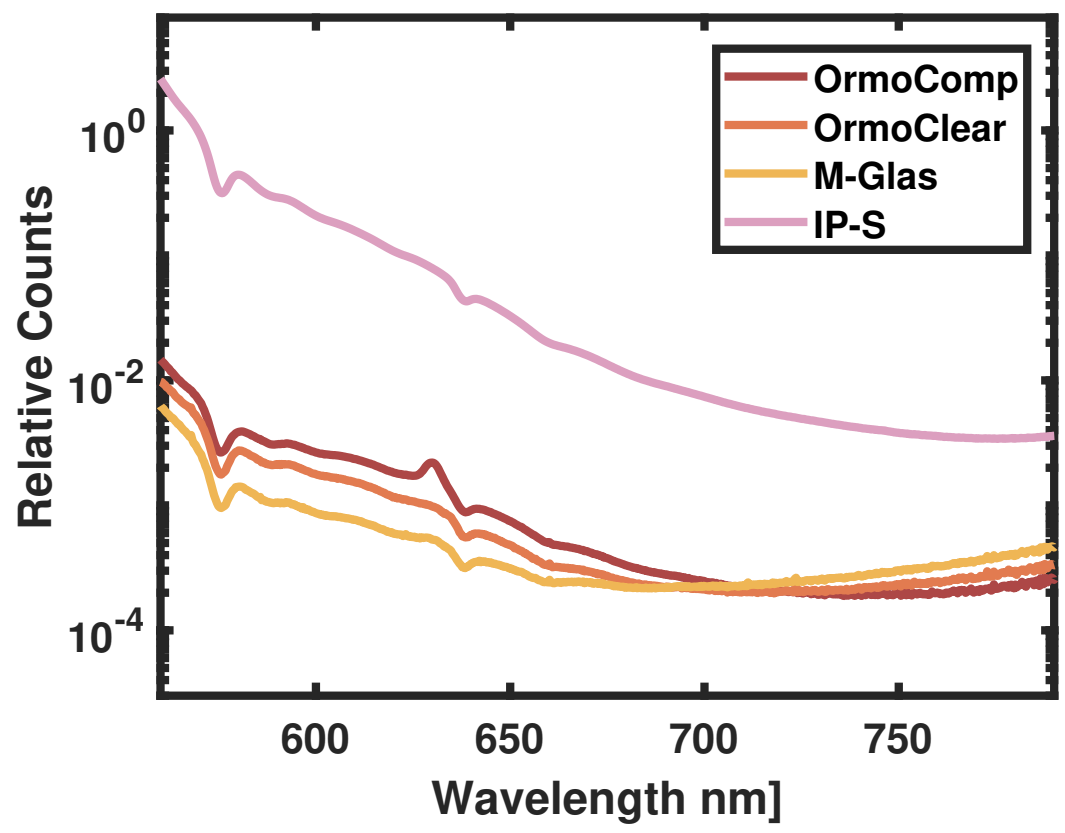

Fig. S12: Spectral comparison of adhesives used in the diamond sensor-head assembly. Measured relative spectrum of the different adhesives used compared to the measured diamond spectrum. The IP-S adhesive showed large auto-fluorescence and was disregarded from further use in the setup.

B.3 Microwave crosstalk

The single magnetometer is expanded into a so-called gradiometer, consisting of two identical sensor heads. This allows to cancel background noise and therefore detect small signals even in everyday environments. This is especially helpful to move the bio-magnetic sensors out of heavy shielded rooms. However, for the gradiometer the two diamonds need to be close together, which means the microwave field from one sensor head might propagate to the other. Simulations showed that at a working distance of 40 mm the leakage to the second diamond sensor is approximately 1.5 % (Fig. S13a black curve). This is a large enough disturbance to interfere with the measurement, especially since later measurements showed that the leakage was much larger than expected from the simulation (see Fig. S13b black and yellow). This is most likely due to a weaker driving frequency of the tested sensor head and a slightly closer proximity of the test antenna. To prevent this cross-talk a shielding around the sensor head is necessary. For testing purposes a household tin-foil (layer thickness of 20 µm), as readily available and easily shaping material, was able to reduce the leakage by one order of magnitude both in simulation

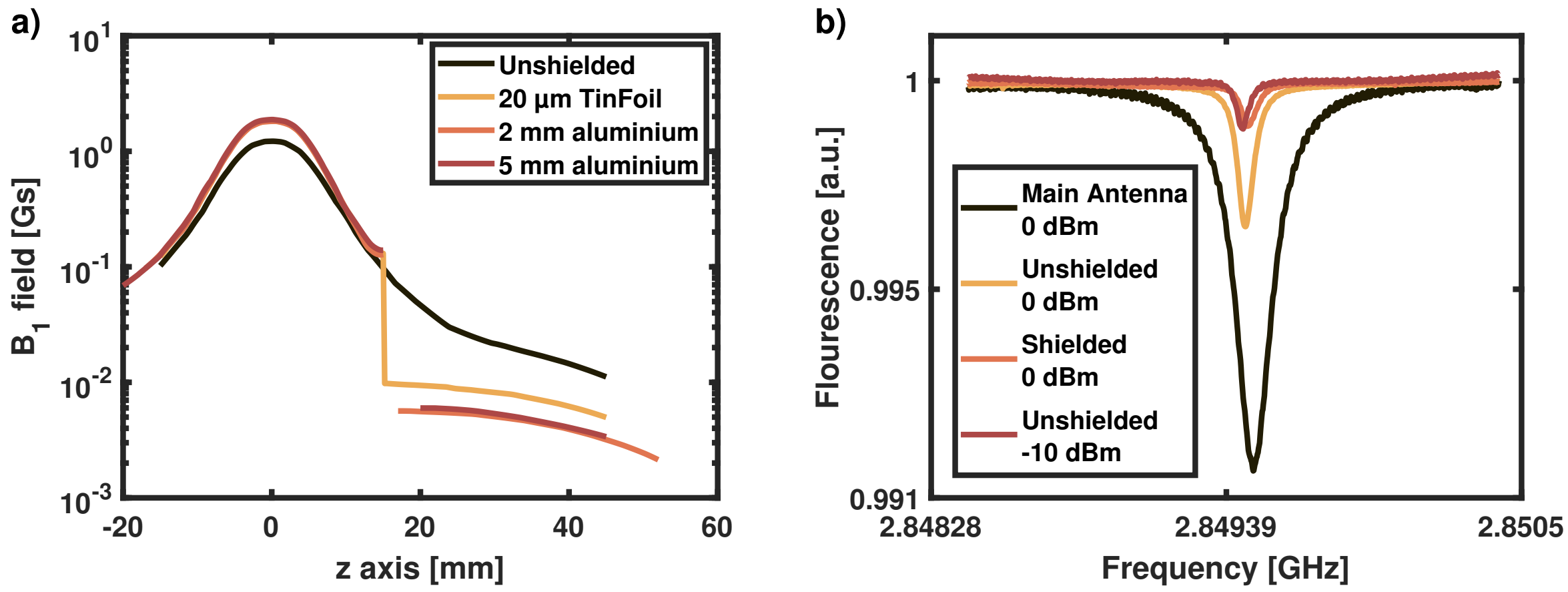

Fig. S13: Simulation and measurement of microwave shielding between gradiometer sensor heads. (a) Simulated microwave field strength along the connecting axis between the two diamonds of the gradiometer with different shieldings. The first sensor head sits at z = 0 mm the second one will be placed at z = 40 mm. The gap in the shielded data is a result of the software, which does not compute the propagation of the electromagnetic waves in the material itself. (b) Measured shielding effect. For reference the spectrum produced by the main antenna of the magnetometer is shown in black. A second microwave antenna producing the MW field for all other measurements was placed 4 cm away. To estimate the reduction the unshielded signal strength was lowered until the amplitude matched the shielded signal.

and measurement, while not interfering with the low-frequency bio-signals. To measure the decreased of the microwave amplitude with a shielding we first measured with the second antenna placed 4 cm away with 0 dBm of microwave power, this clearly shows the crosstalk behavior. With the shielding around the antenna the amplitude of the signal is reduced as expected. In order to find out the reduction strength the unshielded signal is measured once again, however, with a reduced output strength. For a value of −10 dBm the signal amplitude approximately matches the amplitude of the shielded signal. The simulation shows that a shielding out of thicker aluminum can further reduce the crosstalk effect. Thicker casings of course introduce a larger distance between sensor and measurement sample, reducing the effective sensitivity. One also needs to carefully re-assess the effect of a thicker shielding on bio-magnetic signals. The exact crosstalk amount also depends on the final sensor distances, for the here used placement previous analysis from (*57*) was assumed. In a real measurement the optimal distance depends on signal strength and background homogeneity and potentially allows for the use of smaller shieldings. In the future this test shielding can also be expanded into a full enclosure directly solving the third key feature as discussed above by preventing leakage of any laser light to the outside.

B.4 Sensitivity optimization

The fundamental limitation for the sensitivity in CW-ODMR magnetometry is given by the shot noise limit defined as

$$S = P_F \frac{h}{g\mu_B} \frac{\Delta\nu}{C\sqrt{R}}, \tag{S1}$$

where $\Delta\nu$ is the linewidth, C the contrast and R the photon count rate of a single spin transition given by the ODMR spectrum. $P_F = 4/(3\sqrt{3})$ is a prefactor determined by the line profile of

the transition, which here is Lorentzian. To achieve the optimal sensitivity the three parameters need to be optimized and are mainly dependent on the laser and the microwave excitation power. While a larger excitation power means that more photons are collected, due to power broadening the linewidth also increases. Similarly, both the contrast as well as the linewidth increases for a higher microwave power. For the optimal sensitivity those two effects need to be balanced.

In order to convert the measured voltage by the photodiode into a photon rate the output voltage is calibrated beforehand using the laser excitation as reference points to a fluorescence power. In general the quantum efficiency of the photodiode sensor at the different wavelengths needs to be accounted for but is similar enough for the laser and NV fluorescence to be neglected. The thereby obtained fluorescence power then is converted into a photon count rate using

$$R_{\text{photon}} = \frac{P_{\text{FL}}}{E_{\text{photon}}}, \tag{S2}$$

where $E_{\text{photon}} = h \cdot f$ is energy of a single photon with frequency $f = c/\lambda$. For simplicity a single wavelength of 700 nm is assumed, which leads to a slight underestimation of the actual photon number and therefore a worse sensitivity than in reality.

While the laser and microwave power are usually well defined parameters they cannot be measured independently of external factors in our setup, such as light coupling efficiency for the laser power and losses at all electronic parts for the microwave. Therefore, we define a new set of parameters to ensure comparability between measurements of different setups. The chosen parameters here are the Rabi frequency and the optical pumping rate. The conversion between the microwave input power P_{mw} (in dBm) and the Rabi frequency Ω_{Rabi} is given by (*58*)

$$\Omega_{\text{Rabi}} = \sqrt{A \cdot 10^{\left(\frac{B}{10} \cdot P_{\text{MW}}\right)}}, \tag{S3}$$

where A and B are fitting parameters. For the optimization here the corresponding conversion between microwave power at the generator and the Rabi frequency is shown in Fig. S14b. The slowest Rabi oscillations cannot be measured experimentally as the decay is faster than the oscillation and is therefore estimated by extrapolating the fitted curve.
The pumping rate provides a way to calibrate the set laser power to the absorbed power by all NV centers. One difficulty in our large ensemble measurements compared to single NV or small ensemble studies is that the pumping rate cannot be increased enough to achieve saturation. This means the usual approach to derive the optical pumping rate by a saturation curve cannot be used. Instead we use a pulsed microwave system to measure the spin decoherence and infer the pumping rate. The polarization rate and therefore the pumping rate is then given by a polynomial of the form

$$\text{Pumping Rate} = L \cdot \text{Polarization rate} = \frac{L}{T_{1,cw}} = L \cdot (A\,(P_{\text{laser}})^p + c), \tag{S4}$$

where A, p and c are again fitting parameters. $L = 2.76$ is the conversion factor between the polarization rate and the optical pumping rate. In general the relation between the two parameters is non-linear, but for the low laser powers and according low pumping rates in this work a constant connection can be assumed. Details about the calculation of the conversion factor can be found in (*59*). Note that the measured spin decoherence time $T_{1,cw}$ refers to a measurement under constant laser pumping. The theoretical polarization rate without any laser excitation c for this particular diamond is measured to be 0.16 kHz and is fixated here. For the optimization outlined here the corresponding conversion between laser excitation power, measured before the sensor stack, and the pumping rate is shown in Fig. S14c.

To obtain the optimal parameters ODMR sweeps for different Rabi frequencies and pumping rates are carried out. Each spectrum is fitted using a single Lorentzian function of the form

$$\text{Counts}(f) = \frac{A}{\pi} \cdot \frac{\frac{1}{2} \cdot \Delta\nu}{(f - f_0)^2 + (\frac{1}{2} \cdot \Delta\nu)^2} + R_0. \tag{S5}$$

The linewidth $\Delta\nu$ is directly extracted and the contrast C is given by

$$C = \frac{1}{R_0} \cdot \left| \frac{A}{\pi \cdot \frac{\Delta\nu}{2}} \right|. \tag{S6}$$

This provides the maps shown in Fig. S14d and Fig. S14e for the linewidth and contrast. From this the power broadening of both the microwave and laser can be directly seen. While the laser broadening is minuscule with only 10 % over the measured range, the broadening by the microwave is much more significant with an up to 2.5 larger linewidth. From the full optimization map (Fig. S14f) a clear optimum of the shot-noise limited sensitivity is obtained, reaching $(1.80 \pm 0.07)\,\text{pT}/\sqrt{\text{Hz}}$. When factoring in that only a single NV-axis is probed an optimum of $0.74\,\text{pT}/\sqrt{\text{Hz}}$ can be expected when measurements with four overlapping NV-axis is performed. Note that the optimal sensitivity is achieved when operating in double resonance mode (DR) and driving the hyperfine (HF) transitions simultaneously.

In the DR scheme both spin transitions ($m_s = \pm 1$) are driven simultaneously leading to an increase in contrast of the ODMR spectrum, while the linewidth is only slightly deteriorated. For a given pumping power this increases the proportionality factor of $C/\Delta\nu$ by a factor of 1.5 ± 0.1 as can be seen in Fig. S15a. Theoretically the improvement by using the DR scheme can be calculated from the increase of the contrast at the optimal position with the maximal scalar factor and considering a three-level system as outlined in the calculation of Ref.(*45*). Here the optimal position is at the FWHM (Full Width at Half Maximum) where the population in the two-level system is $|0\rangle = 3/4$ and $|+1\rangle = 1/4$. In the three-level system the population is $|0\rangle = 3/5$ and $|\pm 1\rangle = 1/5$. From this population difference the scalar factor increase, or the increase in sensitivity, is 8/5 or 1.60. The measured enhancement is close to this, with reductions due to technical limitations such as perfectly overlapping the two resonances and balancing the microwave powers.
In the HF driving scheme all three ground states of a single NV axis are driven at the same time. This again leads to an increase in contrast and an increase of the proportionality factor by 2.3 ± 0.2 (Fig. S15b). Theoretically, an increase of a factor 3 can be reached, because the three ground states are not coupled and, therefore, do not interact with each other, resulting in a simple addition of the contrast. Possible explanations for the discrepancy between measurement and theory are a non-symmetrical splitting, an imbalanced mixing of the frequencies, the frequency dependence of the microwave power and a coupling of the electrons in the excited state.

For in-situ magnetic measurements it is advantageous to switch from a sweep of the microwave to a fixed frequency and only track the voltage change over time. This provides much faster response times and the possibility to measure signals of higher frequency. The sensitivity is then given by the slope of the ODMR spectrum around the chosen frequency, with an optimum at the position of the FWHM. By utilizing a lock-in detection scheme the microwave frequency is modulated and the differential of the ODMR spectrum is measured. This increases the slope of the spectrum at the cost of dynamic range as showcased in Fig. S15c. Also the optimal point of measurement is shifted to exactly the resonance frequency.
The final improvement to achieve a good sensitivity even in real measurement conditions is the use of a reference measurement scheme to suppress the contribution of the laser noise in the system. By implementing the reference measurement a decrease of the noisefloor by a factor 3.5 and a reduction of noise spikes in the spectrum was achieved.

All these advancements were used to measure the amplitude spectral density and the final MCG as described in the main text.

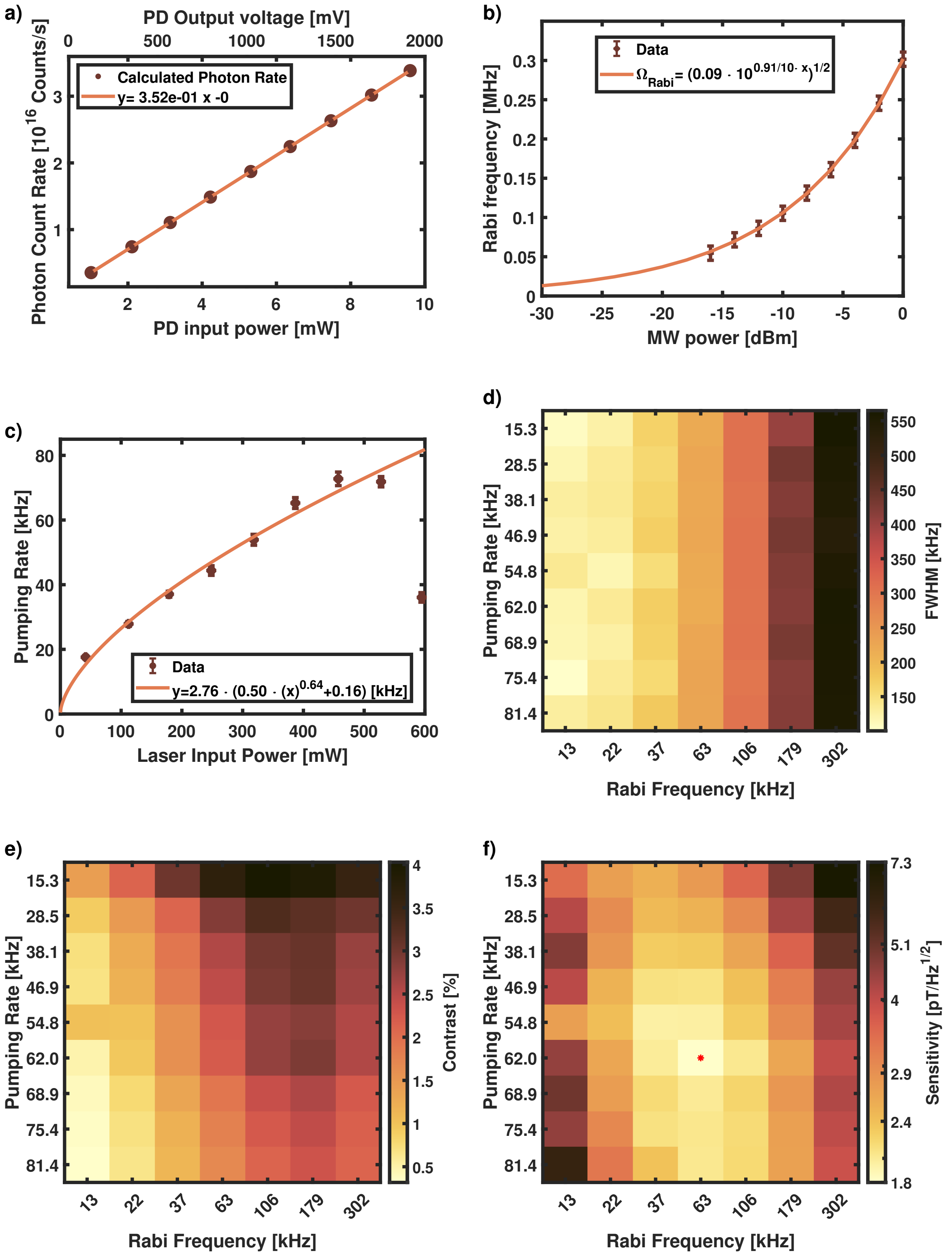

Fig. S14: Microwave- and laser-power optimization for photon-shot-noise-limited sensitivity. Mapping of the microwave and laser power for the optimal photon-shot-noise limited sensitivity. (a) Conversion of the measured photodiode voltage to the photon count rate, the (b) microwave power to the Rabi frequency and the (c) laser power to the polarization rate for an easier comparison between setups. 2D optimization map of the (d) linewidth, (e) contrast and (f) sensitivity in dependence of the Rabi frequency and the polarization rate. The ODMR sweeping utilizes DR as well as hyperfine driving. The best sensitivity is marked with the red dot.

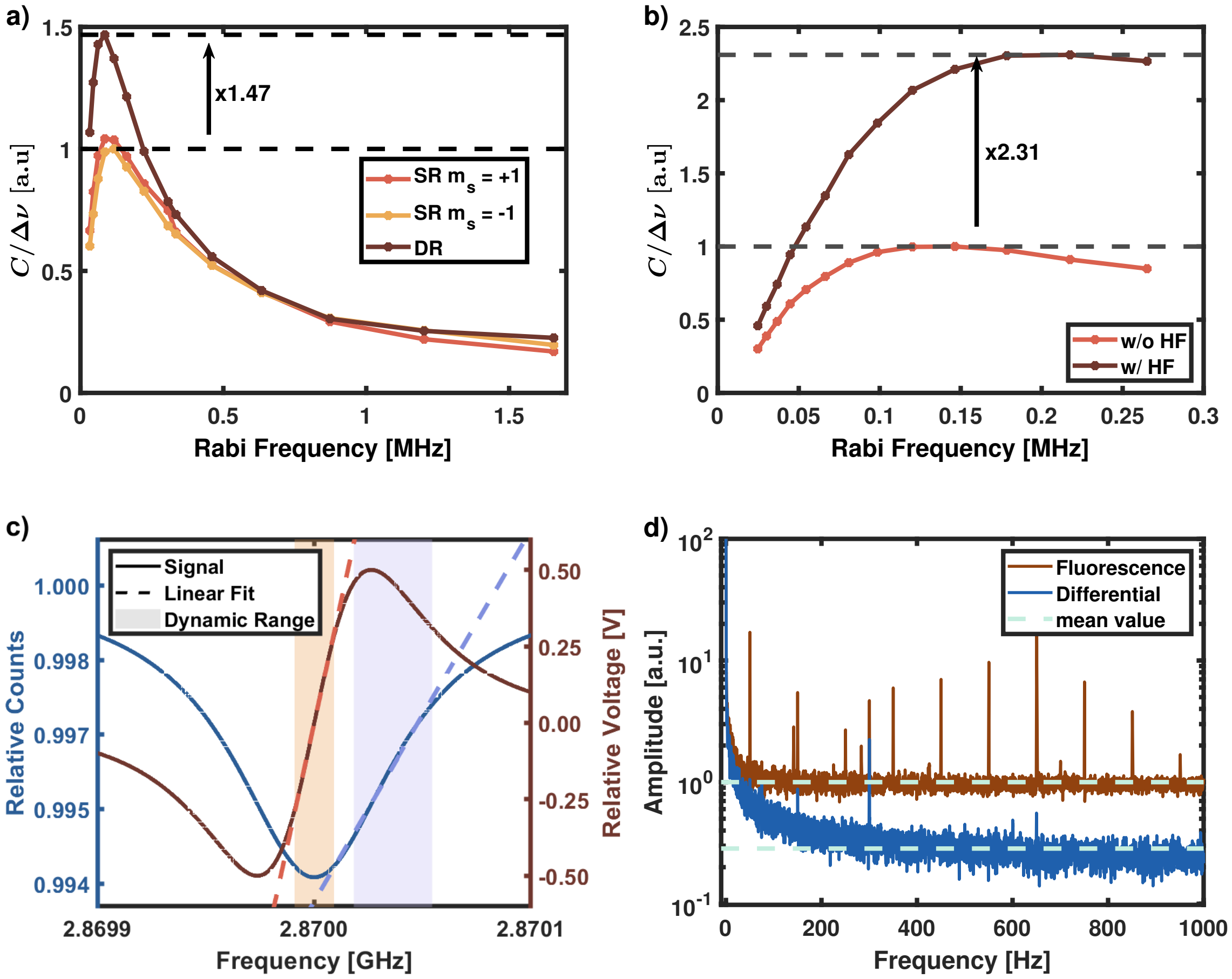

Fig. S15: Methods to increase the sensitivity of the sensor. (a) The double resonance scheme and (b) the hyperfine driving scheme utilize the intrinsic transitions of the NV center by driving both spin states (Double resonance) or all three hyperfine transitions of a single spin state (hyperfine driving) simultaneously. Displayed is the quantity contrast over linewidth $C/\Delta\nu$ for different microwave powers, which defines the sensitivity for a given laser power. (c) Difference between the modulated spectrum obtained by a lock-in measurement scheme and a standard ODMR measurement. The useful region for a magnetic measurement is shaded accordingly. (d) Difference in the amplitude spectral density when utilizing a reference photodiode to reduce the effect of laser fluctuations. Indicated with a dotted line is the mean noisefloor for frequencies >200 Hz compared to the spectrum without any reference.

C Measurement procedures across the three NV magnetometer platforms

All three NV magnetometers were positioned within several centimeters of the chest area. Despite this proximity, extensive signal averaging was required to extract cardiac signatures from the magnetic traces. In all three cases, synchronization for the averaging process was achieved using skin-contact electrodes to record a simultaneous electrocardiogram (ECG), which served as a temporal trigger.

As demonstrated in Fig. S16, the single-shot noise floor of each sensor across its respective sensing bandwidth significantly exceeded the characteristic amplitude of the heart's magnetic signal, precluding real-time detection without post-processing.

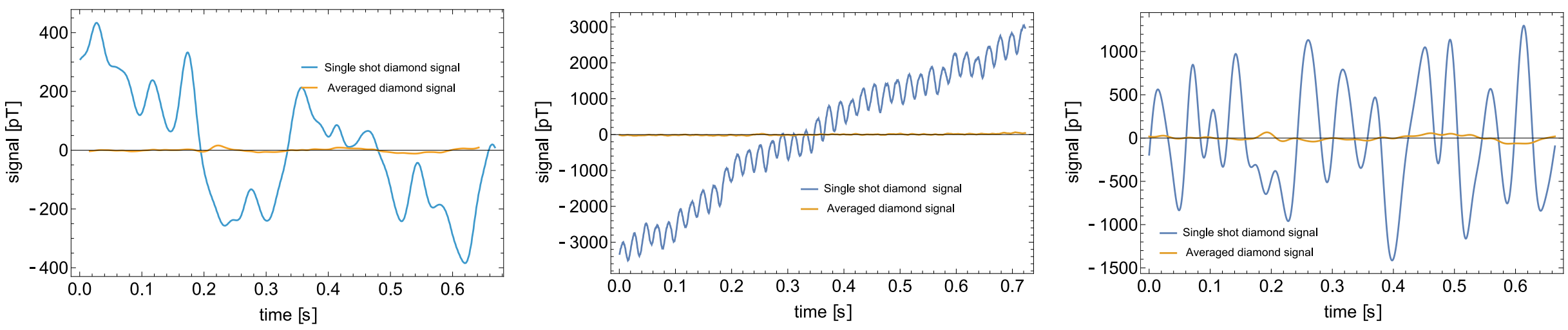

Fig. S16: Comparison of single-shot versus averaged magnetic signal traces across the three platforms: (left) Mainz sensor, (middle) Stuttgart sensor, and (right) Q.Ant sensor.

D Details for blind source separation

D.1 Motivation, typical MCG noise sources, and two-channel simulation model

Gradiometric detection is a standard strategy for suppressing spatially homogeneous magnetic-field noise in biomagnetic measurements. In the simplest implementation, two spatially separated magnetometer channels are subtracted directly,

$$x_{\text{diff}}(t) = x_1(t) - x_2(t), \tag{S7}$$

where $x_1(t)$ and $x_2(t)$ denote the time-dependent magnetic signals recorded by the two sensors. This fixed differential operation is optimal only when the environmental magnetic noise couples identically to both sensors and when the biomagnetic signal is sufficiently localized such that it appears differently in the two channels. In realistic measurements, however, these assumptions are only approximately fulfilled. Small differences in sensor gain, sensor orientation, local shielding response, baseline geometry, and source position can lead to unequal coupling of both biomagnetic and environmental signals into the two channels. In addition, environmental interference in weakly shielded or unshielded environments is often non-stationary and can partially overlap with the cardiac frequency band. Under these conditions, direct subtraction with a fixed subtraction coefficient can leave a residual common-mode component or partially suppress the desired biomagnetic signal.

Practical magnetocardiography (MCG) measurements are further affected by multiple noise sources originating from both the measurement system and the surrounding environment. These include baseline drift, power-line interference, low-frequency $1/f$ noise, white sensor noise, respiratory artifacts, muscle-related artifacts, environmental magnetic disturbances, and transient metallic artifacts. Their approximate physical origins, spectral features, and common mitigation

strategies are summarized in Table S1. Stationary narrowband disturbances, such as power-line interference, can often be reduced using notch filtering or adaptive filtering. However, broadband, non-stationary, or spatially correlated disturbances are more difficult to suppress by filtering alone, especially when their spectral content overlaps with that of the cardiac signal. This motivates the use of multichannel spatial-statistical methods such as blind source separation (BSS), which exploit differences in the spatial coupling and temporal or statistical structure of the underlying sources.

Table S1: **Typical noise components in MCG measurements and commonly used mitigation strategies.**

**Noise component**	**Physical origin and spectral feature**	**Common mitigation strategy**
Baseline drift	Respiration, body motion, sensor–chest distance changes, and sensor drift; typically below 0.5 Hz	High-pass filtering, polynomial fitting, signal averaging
Power-line interference	Power grid, lighting, and hospital equipment; typically 50/60 Hz and harmonics	Notch filtering, adaptive notch filtering
Low-frequency $1/f$ noise	Biological background, geomagnetic fluctuations, and sensor noise; broadband with stronger low-frequency contribution	Wavelet filtering, empirical-mode decomposition, averaging
White noise	Photon shot noise, electronics noise, and thermal noise; broadband	Averaging, low-pass filtering, wavelet thresholding
Respiratory artifact	Chest motion during breathing; typically 0.1–0.5 Hz	Adaptive filtering with respiratory reference, baseline correction
Muscle-related artifact	Skeletal muscle activity and motion artifacts; typically 20–150 Hz	Band-pass filtering, wavelet filtering, ICA/PCA
Environmental magnetic noise	Elevators, vehicles, moving metallic objects, nearby equipment, and geomagnetic fluctuations; variable and often non-stationary	Gradiometry, adaptive filtering, signal-space methods, ICA/BSS
Metallic artifacts	Pacemakers, surgical wires, or nearby moving metallic objects; often low-frequency or transient	ICA, PCA, correlation-based artifact rejection
High-frequency electromagnetic noise	Instrumentation noise and electromagnetic pickup; typically above 100 Hz	Low-pass filtering, shielding, improved grounding

To evaluate BSS-assisted gradiometry under such conditions, we used a two-channel simulation that emulates the situation in which two NV-diamond magnetometers measure different linear mixtures of a weak cardiac magnetic signal and a stronger environmental magnetic-noise contribution. The measured signals are modeled as

$$x_1(t) = a_1 s_{\text{heart}}(t) + b_1 n_{\text{env}}(t) + c\, n_{\text{white},1}(t), \tag{S8}$$

$$x_2(t) = a_2 s_{\text{heart}}(t) + b_2 n_{\text{env}}(t) + c\, n_{\text{white},2}(t), \tag{S9}$$

where $s_{\text{heart}}(t)$ is the cardiac-like magnetic source signal, $n_{\text{env}}(t)$ is the environmental magnetic-noise source, and $n_{\text{white},i}(t)$ is the additive white noise in channel i. The coefficients a_i and b_i describe the spatial coupling strengths of the cardiac and environmental sources to each sensor, while the coefficient c sets the white-noise level.

The coupling coefficients were chosen as

$$a_1 = 1.0, \qquad a_2 = -0.2, \qquad b_1 = 1.0, \qquad b_2 = 0.6. \tag{S10}$$

These values emulate two basic features of a gradiometric MCG configuration. First, the cardiac magnetic field is spatially localized and can therefore exhibit different amplitudes, and even different signs, at the two sensor positions. Second, environmental magnetic noise is more spatially extended and therefore appears in both sensors with the same polarity, although not necessarily with identical amplitude. This unequal environmental coupling is the reason why a fixed subtraction coefficient is not generally optimal.

The simulated source amplitudes were chosen to represent a challenging weak-signal regime. The cardiac-like signal was assigned an amplitude spectral-density scale of approximately 60 $\mathrm{pT}/\sqrt{\mathrm{Hz}}$, whereas the environmental magnetic noise was assigned a larger scale of approximately 1000 $\mathrm{pT}/\sqrt{\mathrm{Hz}}$. The additive white-noise level was set to approximately 0.27 $\mathrm{pT}/\sqrt{\mathrm{Hz}}$. These values are not intended to reproduce one specific experimental dataset, but to test whether the two-channel source-separation procedure can recover a weak biomagnetic component when the directly measured sensor channels are dominated by stronger environmental interference.

To approximate environmental magnetic interference in a realistic weakly shielded or unshielded environment, the environmental noise was modeled as a non-stationary narrowband disturbance with both frequency and amplitude modulation. The instantaneous frequency was defined as

$$f(t) = f_c + \Delta f \sin\left(2\pi f_m t + \phi_f\right), \tag{S11}$$

where $f_c = 12$ Hz is the central frequency, $\Delta f = 8$ Hz is the frequency deviation, $f_m = 0.5$ Hz is the frequency-modulation rate, and ϕ_f is a random initial phase. The amplitude modulation was defined as

$$A(t) = 1 + \alpha \sin\left(2\pi f_a t + \phi_a\right), \tag{S12}$$

where $\alpha = 0.2$ is the modulation depth, $f_a = 0.13$ Hz is the amplitude-modulation frequency, and ϕ_a is a random initial phase.

The environmental noise waveform was then generated as

$$n_{\mathrm{env}}(t) = A(t)\sin[\phi(t)], \tag{S13}$$

where the accumulated phase is

$$\phi(t) = 2\pi \int_0^t f(\tau)\, d\tau. \tag{S14}$$

In the discrete-time implementation, the phase was calculated as

$$\phi[n] = 2\pi \sum_{k=0}^{n} \frac{f[k]}{f_s}, \tag{S15}$$

where f_s is the sampling frequency. The generated environmental noise was finally normalized to zero mean and unit variance according to

$$n_{\mathrm{env}}(t) \leftarrow \frac{n_{\mathrm{env}}(t) - \mu}{\sigma}, \tag{S16}$$

where μ and σ are the mean and standard deviation of the generated time trace, respectively. This construction deliberately creates an environmental disturbance that is more difficult to remove than a stationary sinusoidal interference. Because its instantaneous frequency varies over time, its spectral content is broadened and partially overlaps with the frequency range of the cardiac-like signal. Therefore, simple notch filtering, fixed-band filtering, or fixed-ratio gradiometric subtraction is insufficient to cleanly separate the two components.

D.2 Blind source separation framework

Blind source separation aims to recover latent source signals from multiple measured mixtures without requiring prior knowledge of the exact mixing coefficients or the temporal waveform of each source. For a multichannel measurement, the general linear mixing model can be written as

$$\mathbf{X}(t) = \mathbf{A}\mathbf{S}(t) + \mathbf{n}(t), \tag{S17}$$

where $\mathbf{X}(t) \in \mathbb{R}^m$ is the vector of measured sensor signals, $\mathbf{S}(t) \in \mathbb{R}^r$ is the vector of unknown source signals, $\mathbf{A}$ is the mixing matrix, and $\mathbf{n}(t)$ represents additive measurement noise. In the two-channel case considered here,

$$\mathbf{X}(t) = \begin{bmatrix} x_1(t) \\ x_2(t) \end{bmatrix}. \tag{S18}$$

The objective of BSS is to estimate an unmixing matrix $\mathbf{W}$ such that

$$\mathbf{Y}(t) = \mathbf{W}\mathbf{X}(t), \tag{S19}$$

where $\mathbf{Y}(t)$ contains the recovered source components. In the ideal noiseless case, $\mathbf{W}$ approximates the inverse of the mixing matrix. For the present two-source problem, the recovered components can be interpreted as

$$\begin{bmatrix} y_1(t) \\ y_2(t) \end{bmatrix} = \begin{bmatrix} w_{11} & w_{12} \\ w_{21} & w_{22} \end{bmatrix} \begin{bmatrix} x_1(t) \\ x_2(t) \end{bmatrix}, \tag{S20}$$

where one component corresponds predominantly to the cardiac-like source and the other to the environmental-noise source. Because BSS determines the coefficients from the measured data, it provides a natural way to generalize conventional gradiometry from a fixed subtraction to an adaptive linear recombination of the sensor outputs.
If the recovered cardiac component is written in the form

$$y_{\text{heart}}(t) = w_1 x_1(t) + w_2 x_2(t), \tag{S21}$$

then an equivalent adaptive subtraction coefficient can be defined as

$$\gamma = -\frac{w_1}{w_2}, \tag{S22}$$

after normalizing the coefficient of $x_2(t)$ to unity. Thus, conventional subtraction corresponds to $\gamma = 1$, whereas BSS-assisted gradiometry allows γ to be determined from the data.
Before applying the BSS algorithms, the two measured signals were centered by subtracting their temporal mean:

$$\tilde{\mathbf{X}}(t) = \mathbf{X}(t) - \langle \mathbf{X}(t) \rangle, \tag{S23}$$

where $\langle \cdot \rangle$ denotes temporal averaging. Centering removes the DC offset of each channel and ensures that the source-separation procedure acts on the time-varying components of the measurement.
The covariance matrix of the centered signals is

$$\mathbf{C}_x = \left\langle \tilde{\mathbf{X}}(t) \tilde{\mathbf{X}}^T(t) \right\rangle. \tag{S24}$$

Using the eigenvalue decomposition

$$\mathbf{C}_x = \mathbf{E}\mathbf{D}\mathbf{E}^T, \tag{S25}$$

where $\mathbf{E}$ contains the eigenvectors and $\mathbf{D}$ is the diagonal matrix of eigenvalues, the whitened data are obtained as

$$\mathbf{Z}(t) = \mathbf{D}^{-1/2}\mathbf{E}^T\tilde{\mathbf{X}}(t). \tag{S26}$$

After whitening, the covariance matrix of the transformed data satisfies

$$\left\langle \mathbf{Z}(t)\mathbf{Z}^T(t) \right\rangle = \mathbf{I}. \tag{S27}$$

Whitening normalizes the variance of the measured channels and removes second-order correlations at zero time lag. The remaining source-separation problem is then reduced to finding the rotation that best separates the underlying sources according to the statistical criterion of the chosen BSS algorithm.

D.3 Independent component analysis

Independent component analysis (ICA) separates mixed signals by assuming that the underlying sources are statistically independent and non-Gaussian. After whitening, ICA searches for a rotation of the data that maximizes the statistical independence of the recovered components. In this work, ICA was implemented using the FastICA approach, which estimates the unmixing matrix through an iterative fixed-point procedure.
For a whitened signal vector $\mathbf{z}$, the weight vector $\mathbf{w}$ is updated according to

$$\mathbf{w}^{+} = \left\langle \mathbf{z} g(\mathbf{w}^T\mathbf{z}) \right\rangle - \left\langle g'(\mathbf{w}^T\mathbf{z}) \right\rangle \mathbf{w}, \tag{S28}$$

where $g(\cdot)$ is a nonlinear contrast function and $g'(\cdot)$ is its derivative. A commonly used choice,

$$g(u) = \tanh(u), \tag{S29}$$

was used here. After each update, the weight vector is normalized:

$$\mathbf{w} \leftarrow \frac{\mathbf{w}^{+}}{\|\mathbf{w}^{+}\|}. \tag{S30}$$

This process is repeated until convergence. For the present MCG-like signals, ICA is useful because the cardiac waveform contains temporally localized features that are statistically different from the smoother environmental-noise component.

D.4 Second-order blind identification

Second-order blind identification (SOBI) separates sources using their temporal correlation structure rather than their non-Gaussianity. This is particularly relevant for biomagnetic measurements because both physiological signals and environmental disturbances often exhibit strong temporal correlations.
After centering and whitening, SOBI computes a set of time-delayed covariance matrices,

$$\mathbf{R}_z(\tau_k) = \left\langle \mathbf{Z}(t)\mathbf{Z}^T(t-\tau_k) \right\rangle, \tag{S31}$$

where τ_k denotes the selected time delay. SOBI then searches for an orthogonal matrix $\mathbf{U}$ that approximately jointly diagonalizes these covariance matrices:

$$\mathbf{U}^T\mathbf{R}_z(\tau_k)\mathbf{U} \approx \mathbf{D}_k, \qquad k = 1, 2, \ldots, L, \tag{S32}$$

where each $\mathbf{D}_k$ is approximately diagonal. The recovered source components are then

$$\mathbf{Y}(t) = \mathbf{U}^T\mathbf{Z}(t), \tag{S33}$$

and the corresponding unmixing matrix applied to the centered sensor data is

$$\mathbf{W} = \mathbf{U}^T\mathbf{D}^{-1/2}\mathbf{E}^T. \tag{S34}$$

Because SOBI relies on differences in autocorrelation structure, it can separate sources even when their distributions are not strongly non-Gaussian.
The performance of SOBI depends on the choice of time delays used to construct the delayed covariance matrices. To select suitable lag values, the normalized cross-correlation between the two simulated sensor channels was calculated. The dominant peak near zero delay reflects the strong shared environmental-noise component present in both channels, while additional correlation features at finite delays arise from the temporal structure of the non-stationary interference and cardiac-like signal. The selected SOBI lag values were

$$\tau_{\mathrm{SOBI}} = \begin{bmatrix} 0.032, & 0.081, & 0.130, & 0.208, & 1.793, & 1.870, & 1.919, & 1.968, \\ 2.000, & 2.032, & 2.081, & 2.130, & 2.208, & 3.919, & 3.968, & 4.000, \\ 4.032, & 4.081, & 5.919, & 5.968, & 6.000, & 6.032, & 6.081, & 7.919, \\ 7.968, & 8.000, & 8.032, & 8.081, & 10.000, & 12.000, & 14.000, & 16.000 \end{bmatrix} \mathrm{s}. \quad \text{(S35)}$$

These lags sample both short-time correlations and longer-time correlation features. This combination improves the ability of SOBI to distinguish the cardiac-like waveform from the non-stationary environmental-noise component.

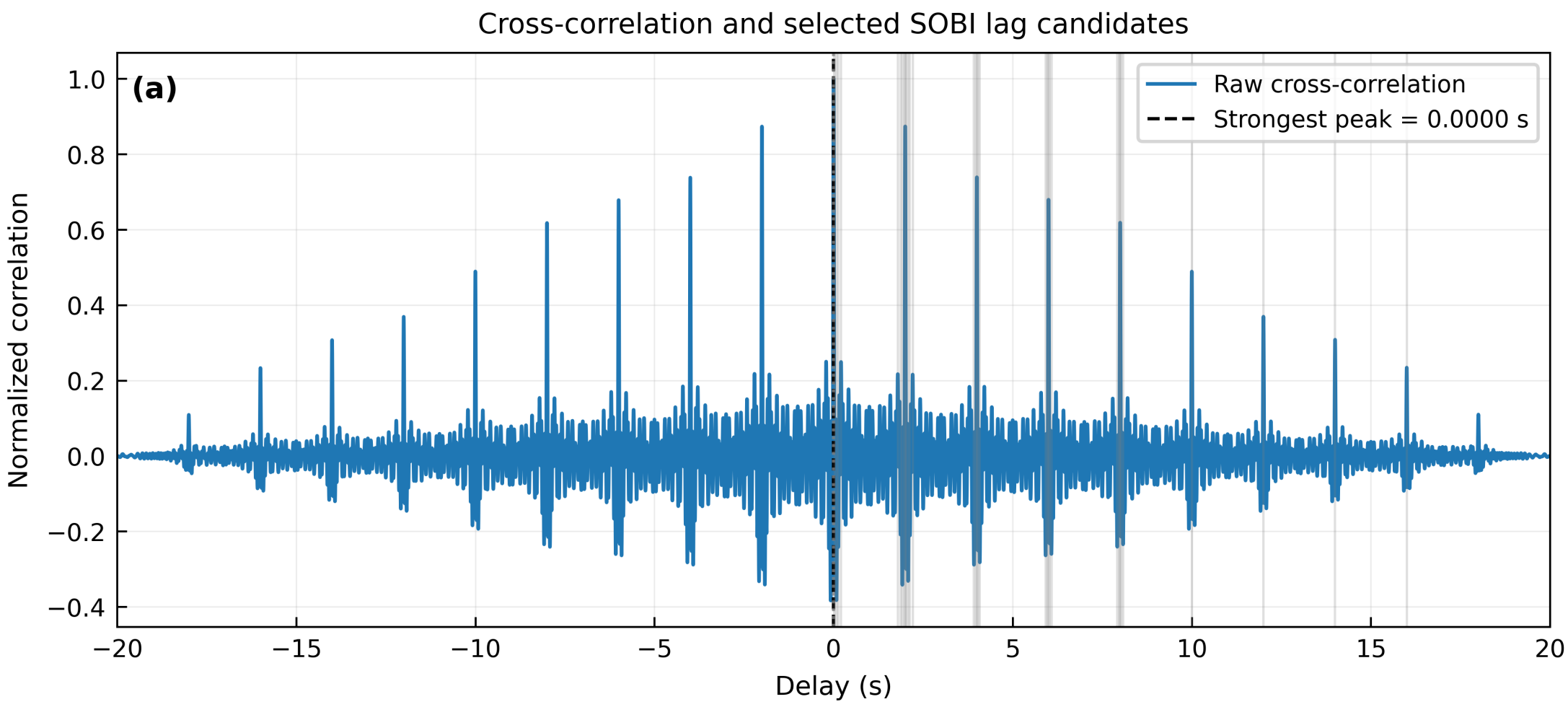

Fig. S17: Normalized cross-correlation between the two simulated NV-magnetometer channels used for the selection of SOBI time delays. The strong peak near zero delay indicates the presence of a dominant shared component in the two channels. The gray vertical lines indicate the lag values used to construct the delayed covariance matrices for SOBI.

D.5 Recovered unmixing matrices and performance evaluation

For the simulated two-channel dataset, the estimated SOBI unmixing matrix was

$$\mathbf{W}_{\mathrm{SOBI}} = \begin{bmatrix} 0.0004 & -0.0024 \\ -0.0124 & 0.0205 \end{bmatrix}. \quad \text{(S36)}$$

For comparison, the ICA-estimated unmixing matrix was

$$\mathbf{W}_{\mathrm{ICA}} = \begin{bmatrix} -0.0002 & -0.0013 \\ 0.0124 & -0.0206 \end{bmatrix}. \quad \text{(S37)}$$

The absolute scale and sign of BSS-recovered components are arbitrary, because multiplying one recovered source by a constant factor or changing its sign does not change the quality of source separation. Therefore, the physical interpretation is based on the waveform shape, spectral

content, and correlation with the known simulated ground-truth sources rather than on the absolute scale of the matrix elements.

From the recovered BSS weights, the corresponding adaptive subtraction coefficient can be inferred by normalizing the recovered cardiac component to the form

$$y_{\mathrm{heart}}(t) \propto x_2(t) - \gamma x_1(t). \tag{S38}$$

In the present simulation, the optimized coefficient is approximately

$$\gamma \approx 1.6, \tag{S39}$$

rather than the fixed value $\gamma = 1$ used in conventional direct subtraction. This result confirms that unequal environmental-noise coupling between the two sensor channels makes simple subtraction suboptimal, and that BSS can identify a more appropriate data-driven linear combination.

The separation performance was evaluated by comparing the recovered components with the known ground-truth cardiac source. As a quantitative metric, we used the Pearson correlation coefficient,

$$\rho_{y,s} = \frac{\sum_t [y(t) - \bar{y}][s_{\mathrm{heart}}(t) - \bar{s}_{\mathrm{heart}}]}{\sqrt{\sum_t [y(t) - \bar{y}]^2 \sum_t [s_{\mathrm{heart}}(t) - \bar{s}_{\mathrm{heart}}]^2}}, \tag{S40}$$

where $y(t)$ is either a measured sensor signal or a recovered BSS component, and bars denote temporal averages. The resulting correlations were

```
corr(x1, heart)      = 0.055
corr(x2, heart)      = -0.025
corr(SOBI-2, heart)  = 0.988
corr(ICA-2, heart)   = 0.990
```

The raw sensor channels show very weak correlation with the cardiac source because they are dominated by the stronger environmental-noise component. In contrast, both SOBI and ICA recover a component with a correlation coefficient close to unity with respect to the ground-truth cardiac signal. This demonstrates that the cardiac-like component can be recovered even when it is not visually apparent in either raw sensor channel.

The power spectral densities of the mixed and recovered signals further illustrate the effect of BSS. Before source separation, the spectra of both sensor channels are dominated by the environmental-noise contribution. After BSS, the recovered components separate the cardiac-like signal from the environmental-noise-dominated component. The white-noise floor is also reduced in the recovered cardiac component because the BSS operation forms an optimized linear combination of the two channels, thereby rejecting the component that is not consistent with the recovered source structure.

REFERENCES

1. J. S. Kwonga, B. Leithäuserb, J.-W. Park, C.-M. Yu, Diagnostic value of magnetocardiography in coronary artery disease and cardiac arrhythmias: A review of clinical data. *Int. J. Cardiol.* **167**, 1835–1842 (2013).

2. R. Fenici, D. Brisinda, A. M. Meloni, Clinical application of magnetocardiography. *Expert Rev. Mol. Diagn.* **5**, 291–313 (2005).

3. M. Oogane, K. Fujiwara, A. Kanno, T. Nakano, H. Wagatsuma, T. Arimoto, S. Mizukami, S. Kumagai, H. Matsuzaki, N. Nakasato, Y. Ando, Sub-pt magnetic field detection by tunnel magneto-resistive sensors. *Appl. Phys. Exp.* **14**, 123002 (2021).

4. H. Karo, K. Shimoda, Y. Maeda, I. Sasada, The first 36 channel fluxgate-sensor-array for the mcg measurement. *IEEJ Trans. Sens. Micromach.* **136**, 224–228 (2016).

5. X. Konga, S. Zhanga, Y. Wanga, J. Zenga, X. Xie, Multi-channel magnetocardiography system based on low-tc squids in an unshielded environment. *Phys. Proc.* **36**, 286–292 (2012).

6. W. Xiao, C. Sun, L. Shen, Y. Feng, M. Liu, Y. Wu, X. Liu, T. Wu, X. Peng, H. Guo, A movable unshielded magnetocardiography system. *Sci. Adv.* **9**, eadg1746 (2023).

7. Y. Yan, J. Lu, S. Zhang, F. Lu, K. Yin, K. Wang, B. Zhou, G. Liu, Three-axis closed-loop optically pumped magnetometer operated in the serf regime. *Opt. Express* **30**, 18300–18309 (2022).

8. C. Kiehl, T. S. Menon, D. P. Hewatt, S. Knappe, T. Thiele, C. A. Regal, Correcting heading errors in optically pumped magnetometers through microwave interrogation. *Phys. Rev. Appl.* **22**, 014005 (2024).

9. M. Tian, L. Jiang, X. Zhao, Y. Chai, J. Liu, Z. Lu, W. Quan, Dead-zone suppression method of nmor atomic magnetometers based on alignment and orientation polarization. *Sens. Actuators A Phys.* **379**, 115842 (2024).

10. J. H. Shim, S.-J. Lee, S. Ghimire, J. I. Hwang, K.-G. Lee, K. Kim, M. J. Turner, C. A. Hart, R. L. Walsworth, S. Oh, Multiplexed sensing of magnetic field and temperature in real time using a nitrogen vacancy spin ensemble in diamond. *Phys. Rev. Appl.* **17**, 014009 (2022).

11. S. Knauer, J. P. Hadden, J. G. Rarity, In-situ measurements of fabrication induced strain in diamond photonic-structures using intrinsic colour centres. *npj Quant. Inform* **6**, 50 (2020).

12. F. Dolde, H. Fedder, M. Doherty, Electric-field sensing using single diamond spins. *Nat. Phys* **7**, 459–463 (2011).

13. J. F. Barry, M. H. Steinecker, S. T. Alsid, J. Majumber, L. M. Pham, M. F. O'Keeffe, D. A. Braje, Sensitive ac and dc magnetometry with nitrogen-vacancy-center ensembles in diamond. *Phys. Rev. Applied* **22**, 044069 (2024).

14. V. V. Soshenko, S. V. Bolshedvorskii, O. Rubinas, V. N. Sorokin, A. N. Smolyaninov, V. V. Vorobyov, A. V. Akimov, Nuclear spin gyroscope based on the nitrogen vacancy center in diamond. *Phys. Rev. Lett.* **126**, 197702 (2021).

15. J. F. Barry, J. M. Schloss, E. Bauch, M. J. Turner, C. A. Hart, L. M. Pham, R. L. Walsworth, Sensitivity optimization for nv-diamond magnetometry. *Rev. Mod. Phys.* **92**, 015004 (2020).

16. I. Fescenko, A. Jarmola, I. Savukov, P. Kehayias, J. Smits, J. Damron, N. Ristoff, N. Mosavian, V. M. Acosta, Diamond magnetometer enhanced by ferrite flux concentrators. *Phys. Rev. Res.* **2**, 023394 (2020).

17. W. C. Griffith, R. Jimenez-Martinez, V. Shah, S. Knappe, J. Kitching, Miniature atomic magnetometer integrated with flux concentrators. *Appl. Phys. Lett.* **94**, 023502 (2009).

18. K. Arai, A. Kuwahata, D. Nishitani, I. Fujisaki, R. Matsuki, Y. Nishio, Z. Xin, X. Cao, Y. Hatano, S. Onoda, C. Shinei, M. Miyakawa, T. Taniguchi, M. Yamazaki, T. Teraji, T. Ohshima, M. Hatano, M. Sekino, T. Iwasaki, Millimetre-scale magnetocardiography of living rats with thoracotomy. *Commun. Phys.* **5**, 200 (2022).

19. Z. Yu, Y. Xie, G. Jin, Y. Zhu, Q. Zhang, F. Shi, F.-Y. Wan, H. Luo, A.-H. Tang, X. Rong, Noninvasive magnetocardiography of a living rat based on a diamond quantum sensor. *Phys. Rev. Appl.* **21**, 064028 (2024).

20. H. Zheng, J. Xu, G. Z. Iwata, T. Lenz, J. Michl, B. Yavkin, K. Nakamura, H. Sumiya, T. Ohshima, J. Isoya, J. Wrachtrup, A. Wickenbrock, D. Budker, Zero-field magnetometry based on nitrogen-vacancy ensembles in diamond. *Phys. Rev. Appl.* **11**, 064068 (2019).

21. M. Omar, J. Xu, R. Kircher, P. Sharbati, S. Zhang, G. Chatzidrosos, J. Eills, R. Picazo-Frutos, D. Budker, D. A. Barskiy, A. Wickenbrock, Zero- to low-field j-spectroscopy with a diamond magnetometer. arXiv: 2512.05776 [physics.app-ph] (2026).

22. I. Altarev, E. Babcock, D. Beck, M. Burghoff, S. Chesnevskaya, T. Chupp, S. Degenkolb, I. Fan, P. Fierlinger, A. Frei, E. Gutsmiedl, S. Knappe-Grüneberg, F. Kuchler, T. Lauer, P. Link, T. Lins, M. Marino, J. McAndrew, B. Niessen, S. Paul, G. Petzoldt, U. Schläpfer, A. Schnabel, S. Sharma, J. Singh, R. Stoepler, S. Stuiber, M. Sturm, B. Taubenheim, L. Trahms, J. Voigt, T. Zechlau, A magnetically shielded room with ultra low residual field and gradient. *Rev. Sci. Instrum.* **85**, 075106 (2014).

23. M. Omar, A. Conta, A. Westerhoff, R. Hasse, G. Chatzidrosos, D. Budker, A. Wickenbrock, Diamond-optic enhanced photon collection efficiency for sensing with nitrogen-vacancy centers. *Opt. Lett.* **48**, 2512–2514 (2023).

24. G. Heinzel, A. Rüdiger, R. Schilling, "Spectrum and spectral density estimation by the discrete fourier transform (dft), including a comprehensive list of window functions and some new at-top windows," *Max-Planck-Institut für Gravitationsphysik Report* (2002).

25. M. Omar, M. Benke, S. Zhang, J. Zhang, Y. Wang, M. Kuebler, P. Sharbati, A. Rahimpour, A. Gück, M. Kapitonova, D. Kadam, C. R. Izquierdo Geiser, J. Haller, A. Trautmann, K. Jag-Lauber, R. Rölver, T.-D. Nguyen, L. Gizzi, M. Schweizer, M. Abdelsayed, I. Wickenbrock, A. M. Edmonds, M. Markham, P. A. Koss, O. Schnell, U. G. Hofmann, T. Ball, J. Beck, D. Budker, J. Wrachtrup, A. Wickenbrock, Supplementary materials for "human cardiac measurements with diamond magnetometers" (2026). Available as supplementary materials accompanying this manuscript.

26. Task Force of the European Society of Cardiology and the North American Society of Pacing and Electrophysiology, Heart rate variability. Standards of measurement, physiological interpretation, and clinical use. *Eur. Heart J.* **17**, 354–381 (1996).

27. S. E. Robinson, A. B. Andonegui, T. Holroyd, K. J. Hughes, O. Alem, S. Knappe, T. Maydew, A. Griesshammer, A. Nugent, Cross-axis dynamic field compensation of optically pumped magnetometer arrays for meg. *Neuroimage* **262**, 119559 (2022).

28. S. Su, Z. Xu, X. He, G. Zhang, H. Wu, Y. Gao, Y. Ma, C. Yin, Y. Ruan, K. Li, Q. Lin, Vector magnetocardiography using compact optically-pumped magnetometers. *Heliyon* **10**, e29092 (2024).

29. W. Zheng, S. Su, G. Zhang, X. Bi, Q. Lin, Vector magnetocardiography measurement with a compact elliptically polarized laser-pumped magnetometer. *Biomed. Opt. Express* **11**, 649–659 (2020).

30. Y. J. Kim, I. Savukov, S. Newman, Magnetocardiography with a 16-channel fiber-coupled single-cell rb optically pumped magnetometer. *Appl. Phys. Lett.* **114**, 143702 (2019).

31. A. L. Goldberger, Z. D. Goldberger, A. Shvilkin, *Goldberger's Clinical Electrocardiography: A Simplified Approach* (Elsevier, ed. 9, 2017).

32. S. Taulu, J. Simola, J. Nenonen, L. Parkkonen, *Novel Noise Reduction Methods* (Springer), 35–71 (2014).

33. O. Alem, K. J. Hughes, I. Buard, T. P. Cheung, T. Maydew, A. Griesshammer, K. Holloway, A. Park, V. Lechuga, C. Coolidge, M. Gerginov, E. Quigg, A. Seames, E. Kronberg, P. Teale, S. Knappe, An integrated full-head opm-meg system based on 128 zero-field sensors. *Front. Neurosci.* **17**, 1190310 (2023).

34. P. P. Swain, S. Sengottuvel, R. Patel, A. Mani, K. Gireesan, A feasibility study to measure magnetocardiography (MCG) in unshielded environment using first order gradiometer. *Biomed. Signal Process. Control* **55**, 101664 (2020).

35. G. Chatzidrosos, A. Wickenbrock, L. Bougas, N. Leefer, T. Wu, K. Jensen, Y. Dumeige, D. Budker, Miniature cavity-enhanced diamond magnetometer. *Phys. Rev. Appl.* **8**, 044019 (2017).

36. A. Tayefeh Younesi, M. Omar, A. Wickenbrock, D. Budker, R. Ulbricht, Towards high-sensitivity magnetometry with nitrogen-vacancy centers in diamond using the singlet infrared absorption. *Phys. Rev. Appl.* **23**, 054019 (2025).

37. E. R. Eisenach, J. F. Barry, M. F. O'Keeffe, J. M. Schloss, M. H. Steinecker, D. R. Englund, D. A. Braje, Cavity-enhanced microwave readout of a solid-state spin sensor. *Nat. Commun.* **12**, 1357 (2021).

38. H. Wang, K. Jacobs, D. Fahey, Y. Hu, D. R. Englund, M. E. Trusheim, Exceptional sensitivity near the bistable transition point of a hybrid quantum system. arXiv: 2507.09691 [quant-ph] (2025).

39. H. Gao, L. S. Martin, L. B. Hughes, N. T. Leitao, P. Put, H. Zhou, N. U. Koyluoglu, S. A. Meynell, A. C. B. Jayich, H. Park, M. D. Lukin, Signal amplification in a solid-state quantum sensor via asymmetric time-reversal of many-body dynamics. arXiv: 2503.14598 [quant-ph] (2025).

40. W. Wu, E. J. Davis, L. B. Hughes, B. Ye, Z. Wang, D. Kufel, T. Ono, S. A. Meynell, M. Block, C. Liu, Spin squeezing in an ensemble of nitrogen-vacancy centers in diamond. arXiv: 2503.14585 [quant-ph] (2025).

41. Y. Xie, H. Yu, Y. Zhu, X. Qin, X. Rong, C.-K. Duan, J. Du, A hybrid magnetometer towards femtotesla sensitivity under ambient conditions. *Sci. Bull.* **66**, 127–132 (2021).

42. N. Sekiguchi, M. Fushimi, A. Yoshimura, C. Shinei, M. Miyakawa, T. Taniguchi, T. Teraji, H. Abe, S. Onoda, T. Ohshima, M. Hatano, M. Sekino, T. Iwasaki, Diamond quantum magnetometer with dc sensitivity of sub-10 pT $Hz^{-1/2}$ toward measurement of biomagnetic field. *Phys. Rev. Appl.* **21**, 064010 (2024).

43. T. Lenz, A. Wickenbrock, F. Jelezko, G. Balasubramanian, D. Budker, Magnetic sensing at zero field with a single nitrogen-vacancy center. *Quant Sci. Technol.* **6**, 044010 (2021).

44. C. Zhang, F. Shagieva, M. Widmann, M. Kübler, V. Vorobyov, P. Kapitanova, E. Nenasheva, R. Corkill, O. Röhrle, K. Nakamura, H. Sumiya, S. Onoda, J. Isoya, J. Wrachtrup, Diamond magnetometry and gradiometry towards subpicotesla dc field measurement. *Phys. Rev. Appl.* **15**, 064075 (2021).

45. Blender Online Community, Blender, version 4.3.0, Blender Foundation, Blender Download Archive (2024).

46. Inkscape Project, Inkscape, Inkscape Release Archive (2024).

47. A. N. Matlashov, L. J. Schultz, M. A. Espy, R. H. Kraus, I. M. Savukov, P. L. Volegov, C. J. Wurden, Squids vs. induction coils for ultra-low field nuclear magnetic resonance: Experimental and simulation comparison. *IEEE Trans. Appl. Supercond.* **21**, 465–468 (2011).

48. S. I. Bogdanov, M. Y. Shalaginov, A. S. Lagutchev, C.-C. Chiang, D. Shah, A. S. Baburin, I. A. Ryzhikov, I. A. Rodionov, A. V. Kildishev, A. Boltasseva, V. M. Shalaev, Ultrabright room-temperature sub-nanosecond emission from single nitrogen-vacancy centers coupled to nanopatch antennas. *Nano Lett.* **18**, 4837–4844 (2018).

49. A. Karamlou, M. E. Trusheim, D. Englund, Metal-dielectric antennas for efficient photon collection from diamond color centers. *Opt. Express* **26**, 3341–3352 (2018).

50. S. A. Momenzadeh, R. J. Stohr, F. F. De Oliveira, A. Brunner, A. Denisenko, S. Yang, F. Reinhard, J. Wrachtrup, Nanoengineered diamond waveguide as a robust bright platform for nanomagnetometry using shallow nitrogen vacancy centers. *Nano Lett.* **15**, 165–169 (2015).

51. L. Rondin, J.-P. Tetienne, T. Hingant, J.-F. Roch, P. Maletinsky, V. Jacques, Magnetometry with nitrogen-vacancy defects in diamond. *Rep. Prog. Phys.* **77**, 056503 (2014).

52. T. Zhu, J. Rhensius, K. Herb, V. Damle, G. Puebla-Hellmann, C. L. Degen, E. Janitz, Multicone diamond waveguides for nanoscale quantum sensing. *Nano Lett.* **23**, 10110–10117 (2023).

53. H. Clevenson, M. E. Trusheim, C. Teale, T. Schröder, D. Braje, D. Englund, Broadband magnetometry and temperature sensing with a light-trapping diamond waveguide. *Nat. Phys.* **11**, 393–397 (2015).

54. H. Deguchi, T. Hayashi, H. Saito, Y. Nishibayashi, M. Teramoto, M. Fujiwara, H. Morishita, N. Mizuochi, N. Tatsumi, Compact and portable quantum sensor module using diamond nv centers. *Appl. Phys. Exp.* **16**, 062004 (2023).

55. L. Xu, H. Yuan, N. Zhang, J. Zhang, G. Bian, P. Fan, M. Li, C. Zhang, Y. Zhai, J. Fang, High-efficiency fluorescence collection for nv-center ensembles in diamond. *Opt. Express* **27**, 10787–10797 (2019).

56. D. Le Sage, L. M. Pham, N. Bar-Gill, C. Belthangady, M. D. Lukin, A. Yacoby, R. L. Walsworth, Efficient photon detection from color centers in a diamond optical waveguide. *Phys. Rev. B* **85**, 121202(R) (2012).

57. C. Zhang, J. Zhang, M. Widmann, M. Benke, M. Kübler, D. Dasari, T. Klotz, L. Gizzi, O. Röhrle, P. Brenner, J. Wrachtrup, Optimizing NV magnetometry for magnetoneurography and magnetomyography applications. *Front. Neurosci.* **16**, 1034391 (2023).

58. K. Wang, G. Xu, F. Gao, Ultafast coherent control of a hole spin qubit in a germanium quantum dot. *Nat. Commun.* **13**, (2022).

59. J. Zhang, L. Xu, T. Shi, G. Bian, P. Fan, M. Li, P. Chen, C. Xu, N. Zhang, H. Yuan, An improved spin readout for nitrogen vacancy center ensemble based on a maximum likelihood estimation method. *J. Phys. D Appl. Phys.* **53**, 455305 (2020).
